# Functional analyses and integrated mechanisms of cellular destruction by L-amino acid oxidase

**DOI:** 10.1038/s41419-025-08187-7

**Published:** 2025-11-20

**Authors:** Krisna Prak, Christin Luft, Eliona Tsefou, Carlos Chávez-Olórtegui, Janos Kriston-Vizi, Robin Ketteler, Vania M. M. Braga

**Affiliations:** 1 National Heart and Lung Institute, Faculty of Medicine, Imperial College London, London, UK; 2 Laboratory for Molecular Cell Biology, University College London, London, UK; 3 Biochemistry and Immunology Department, Institute of Biological Sciences, Federal University of Minas Gerais, Belo Horizonte, Brazil; 4 Department of Human Medicine, Medical School Berlin, Berlin, Germany

**Keywords:** Cell biology, Diseases

## Abstract

Snakebite accidents are prevalent worldwide and cause a spectrum of severe clinical manifestations and result in a reduction of patient quality of life and economic income. A major bottleneck in envenomation treatment is our limited understanding of how venom toxins perturb specific cellular processes involved in tissue necrosis. Here, we address this knowledge gap and define the cellular mechanisms via which cell death is triggered by the snake toxin L-amino acid oxidase (LAAO). LAAO is a highly toxic enzyme present in various venoms that causes tissue necrosis, edema, coagulopathies, and organ failure. Here, we identify the residues essential for LAAO oxidation and obtain a catalytically inactive LAAO mutant, which is unable to reproduce the cellular phenotypes. Striking cellular defects are triggered by a catalysis-dependent increase in oxidative stress, via H_2_O_2_ (reaction byproduct). LAAO uptake by cells leads to a decrease in lysosome number and size and inhibits autophagy flux. In parallel, mitochondria function is impaired by severe proton leakage, and mitochondrial fission is stimulated, causing their engulfment by autophagosomes. However, mitochondrial clearance is prevented by the lysosomal defects. The concurrent shutdown of cell respiration and energy consumption indicates that LAAO catalysis reduces both metabolism and cell fitness. Thus, essential organelles are coordinately impaired by LAAO activity, accelerating cell demise. Considering the multi-organelle impairment, strategies to reduce organelle injury after LAAO exposure may be effective to maintain critical cell functions and strengthen adaptive responses against cytotoxicity.

## Introduction

Compared to other neglected tropical diseases, snakebites have a similar global prevalence to cholera infection [1] and yet show the largest patient lethality, disproportionately affecting the poorest communities in developing countries [2]. The highest impact of snakebite accidents is seen on the severe, debilitating disabilities that often cause a lifelong reduction of income and quality of life with extensive necrosis, disfigurement, and amputations [1, 3]. Available treatment for envenoming patients relies on quenching of circulating venom toxins with antivenom sera (when available in hospitals and with appropriate snake species specificity) and the natural elimination of toxins from the body. Because of the high costs and low availability of anti-venoms worldwide, there is currently an unmitigated clinical need to develop novel treatments for snakebite envenoming [4, 5], particularly the devastating necrosis at the snakebite site where anti-venoms are not effective.

Venoms are a cocktail of various toxins, and cooperation and interactions among toxins are likely to occur. A concerted effort in the field has been to characterize the structure and biochemistry of the clinically relevant venom toxins and their action in animal models. However, dissecting the function of specific toxins at the cellular level is essential to understand their individual contributions to specific in vivo symptoms at the organism level [6]. Indeed, the cellular perturbations caused by a toxin (i.e., cell death and acute oxidative stress) have the hallmarks of in vivo envenomation phenotypes such as tissue necrosis and organ failure, e.g., [7]. It is clear that (i) the lack of detailed cellular mechanisms of how specific toxins promote cell death is a major hurdle in the field and (ii) identifying the function of a reptile toxin on mammalian cells forms a springboard to understand their complex phenotypes in tissues and organs.

Whether the specific responses elicited are a direct consequence of the action of venom toxins or a defensive mechanism to protect against cytotoxicity is unclear. It is indeed feasible that cell adaptive responses against toxicity may compound the severe reduction in cell fitness and accelerate cell death [8]. Nevertheless, understanding the cellular action of venom toxins may provide insights into regenerating tissue damage and accelerate patient recovery.

To address the above conceptual gap, we performed a structure-function analysis of snake venom L-amino acid oxidase (LAAO, E.C.1.4.3.2) to inform its cellular mechanisms. Purified native venom LAAO causes local tissue damage, altered platelet aggregation, hemorrhage, hemolysis, and edema in animal models [9]. LAAO proteins are found from bacteria to mammals. They have conserved catalytic pockets and are thought to participate in amino acid catabolism and defensive mechanisms [10]. Mammalian LAAOs are found in the brain, milk, sperm and immune cells. Among mammalian LAAOs, Interleukin-4 induced gene 1 (IL4I1) is the best characterized protein; it is secreted at human immune synapses [11] and contributes to immunoregulatory responses. While both human LAAO and snake venom LAAO oxidize L-amino acids, only the venom counterpart is cytotoxic [12].

Snake venom LAAOs are flavoproteins identified in 1979 that have preferential catalytic specificity for hydrophobic and aromatic L-amino acids [9, 13]. After oxidation of L-amino acid substrate, the catalytic byproducts (ammonia and hydrogen peroxide) are toxic *per se in cellulo* and in vivo [14, 15]. Hydrogen peroxide can be converted to reactive oxygen species (ROS), i.e., highly reactive hydroxyl radical or intracellular superoxide, which have major effects in various cellular processes [16]. An attractive possibility that is currently underexplored is whether LAAO cytotoxicity is triggered by alterations in amino acid catabolism and exacerbated by the oxidative stress responses. For example, oxidative stress is a key feature of envenomation by both purified toxins and crude venom from various snake species [14, 17], and preventing acute oxidative stress can minimize localized necrosis in animal models [18].

The specific cellular mechanisms underlying snake venom LAAO toxicity are poorly understood. We found that purified native LAAO is internalized by primary human cells and causes cytoplasmic vesicle swelling, pyknotic nuclei, cell retraction, induction of autophagy, followed by apoptosis and necrosis [18]. Autophagy is a cellular process that regulates catabolism, homeostasis and quality control of organelles [19]. During intoxication by different snake venoms or purified toxins [18, 20], autophagy may be a common denominator of cellular envenomation.

LAAO toxicity [18] likely alters cellular homeostasis processes essential for cell survival. We hypothesize that LAAO catalysis interferes directly or indirectly with the functions of mitochondria and lysosomes, two essential organelles. Mitochondria participate in programmed cell death regulation and have a key function in energy production, ROS generation and ROS metabolism [21, 22]. Mitochondrial quality control is essential for the maintenance of cellular homeostasis: damaged mitochondria are removed via mitophagy to maintain cell fitness [23]. Dysfunctional lysosomes are associated with various pathological status [24] as lysosomes are key regulators of degradative processes, cell secretion, metabolic sensing and adaptation [25].

We envisage three possibilities. First, the catalysis byproduct ammonia may inhibit lysosomal fusion with other organelles [26], while oxidative stress generated by its catalysis could damage mitochondria. Second, LAAO is expected to oxidize and reduce the levels of its amino acid substrates, which may alter cell metabolic rate. Third, it is also feasible that the unbalancing of amino acid levels may affect lysosomal function, as lysosomes form a platform for activation of the amino acid-sensing machinery [27].

To overcome the restricted availability of purified native toxins from snake venoms [18], we produced active recombinant LAAO from *Bothrops atrox* (*B. atrox*) snake, the species responsible for most accidents in the Brazilian Amazon [3, 28]. Available crystal structures of native LAAO from different snake species detail a remarkable domain conservation of a helical domain that provides access to the active site and the binding domains of the cofactor flavin-adenine dinucleotide (FAD) and substrate [15, 29, 30]. Yet, conserved residues that participate in LAAO catalysis [30] have not yet been formally identified (apart from one study) [12].

Using various LAAO mutants and kinetic analyses, we demonstrate unequivocally the key amino acid residues important for catalysis and confirm their contribution in vitro and *in cellulo*. Comprehensive temporal analyses of LAAO-induced cellular phenotypes show that LAAO induces cell death by targeting mitochondrial and lysosomal function and dynamics, and impairs catabolic processes, energy production and consumption. Our data shed insights into the LAAO-dependent early cytotoxic events and provide a platform to investigate the factors underpinning the rapid cell death upon exposure to snake toxins. Our data are instrumental in increasing our conceptual knowledge of toxins on their own right and in identifying strategies to ameliorate cell destruction and preserve tissue integrity.

## Methods

### Plasmid cloning

Wild-type LAAO from *B. atrox* (LAAO^WT^) was inserted into the pcDNA3.1 vector with a His-tag at the C-terminus as described elsewhere (manuscript in preparation). To create point mutations in pcDNA-LAAO^WT^, relevant primers were designed (Table S1) to introduce the following mutations: R90A (LAAO^R90A^), N172A (LAAO^N172A^), H223A (LAAO^H223A^), R322A (LAAO^R322A^) and Y372A (LAAO^Y372A^). We aimed to test the following predictions: R90A mutation is predicted to remove the interaction between LAAO, substrate and cofactor FAD, while N172A abolishes a glycosylation site. H223A and R322A mutations are predicted to interfere with substrate binding sites and Y372A should interfere with both substrate/ligand binding sites. All PCRs were conducted for 30 cycles using Pyrobest DNA polymerase (Takara, R005A). PCR products were phosphorylated and ligated. All clones were verified by DNA sequencing.

### Cell culture

All cells were cultured in a humid chamber at 37 °C and 5% CO_2_. HEK293T (human embryonic kidney cells) were used to produce recombinant LAAO proteins only. They were grown in a 500 cm^2^ large Nunc™ square culture dish (Thermo Scientific, 240835; 100 ml culture media) in DMEM medium containing high glucose (Sigma, D6546) supplemented with GlutaMax (Gibco, 35050-061) and 10% fetal calf serum (FCS; Seralab, EU-000-F) until they reach the confluency of around 80%. The cells were then transfected with 50 µg of plasmids pcDNA-LAAO, pcDNA-LAAO^WT^, pcDNA-LAAO^R90A^, pcDNA-LAAO^N172A^, pcDNA-LAAO^H223A^, pcDNA-LAAO^R322A^ or pcDNA-LAAO^Y372A^ using FuGENE® HD transfection reagent at 1 µg plasmid/4 µl transfection reagent (Promega, E2312).

Normal human epidermal keratinocytes at 4000 cells/well (passages 3–6) were co-cultured with 3T3-J2 fibroblasts in a 96-well plate (Corning, 3585; cell viability assay) or a CellCarrier-96 ultra microplate (PerkinElmer, 6055300; confocal imaging) in FAD standard medium (FAD medium (Gibco, custom-made) as described previously [31]). Where indicated, cells were transfected with pEGFP-LC3 (EGFP-LC3: microtubule-associated protein 1 light chain 3 fused with enhanced green fluorescent protein) (Addgene, 24920) at 0.1 µg of the plasmid (in 50 µl Opti-MEM® Reduced Serum Media) and 0.4 µl of FuGENE® HD Transfection Reagent.

To investigate autophagy flux and mitophagy, two different cell models were used that are well-established in the field: HepG2 (human liver cancer cell line) stably expressing mCherry-EGFP-LC3 and SH-SY5Y (neuroblastoma cell line) cells stably expressing mitochondria-targeted monomeric Keima (mtKeima, MBL, AM-V0251HM) fluorescent protein, respectively. Cells were seeded at 10,000 cells/well in a 384-well plate (Nunc, 164688; cell viability assay or a CellCarrier-384 ultra microplate (PerkinElmer, 6057300; confocal imaging)) in Dulbecco’s modified Eagle’s medium containing GlutaMax (Gibco, 61965-026) supplemented with 10% FCS.

### Recombinant protein production and purification

After transfection for 4 h, the medium was refreshed with medium containing 10 µg/ml catalase (Sigma, C1345) to initiate the collection of recombinant protein produced by the HEK293T secretion system. Medium was collected every 24 h and replaced with fresh medium as described above. After 3 days, the collected media were concentrated 10-fold using Amicon Ultra centrifugal filter units, Ultra-15, MWCO 10 kDa (Sigma, Z706345-8EA). Imidazole at 20 mM (Sigma, 792527-100 G) and 1 mM dithiothreitol were added, and the secreted medium was incubated with 1.5 ml of Ni-NTA Agarose (Qiagen, 30210) for 1 h, shaking at 4 °C for the isolation of recombinant His-tagged LAAO. Beads were centrifuged and washed 5 times with PBS buffer pH 7.3 (140 mM NaCl, 2.7 mM KCl, 10 mM Na_2_HPO_4_, 1.8 mM KH_2_PO_4_) containing 20 mM imidazole and 1 mM DTT. LAAO^WT^ and mutants were eluted twice with 2 ml PBS buffer pH 7.3 containing 150 mM imidazole and 1 mM DTT. The proteins were concentrated to 1 ml and washed with 50 mM sodium acetate buffer, pH 5.0, containing 150 mM NaCl and 1 mM DTT. For long-term storage, glycerol was added (10% volume/volume), and the proteins were aliquoted and stored at −80 °C.

Protein concentrations were determined by loading different amounts of recombinant LAAO on a 10% sodium dodecyl sulfate (SDS)–polyacrylamide gel electrophoresis and staining with Coomassie Blue. Different concentrations of bovine serum albumin (BSA) (Thermo Fisher, 23209) were loaded as a standard in each gel. Band intensities were quantified using Fiji and the apparent concentration of recombinant LAAO derived from the corresponding BSA standard curve.

### Activity assays and enzyme kinetics of LAAO^WT^ and mutants

Enzymatic assay for LAAO activity was conducted with modifications from what was described [32]. To determine LAAO activity in the secreted media, 30 µl of secreted medium and 70 µl of LAAO assay solution (0.1 M Tris-HCl pH 8.0, 5 mM L-leucine, 2 mM OPD (o-phenylenediamine dihydrochloride) (Sigma, P9187), 33.3 µg/ml peroxidase (Sigma, 77332)), were added to a 96-well ELISA plate (Thermo Fisher, 612U96) and incubated for 1 h at 37 °C. The reaction was stopped by adding 50 µl of 1 M sulfuric acid (H_2_SO_4_). The absorbance was determined at 490 nm using a SpectraMax i3X and Pro 6.5 software.

To investigate the activity of purified recombinant LAAO^WT^ and mutants in the presence of excessive substrate L-leucine (5 mM), 52.72 nM (or 0.3 µg per 100 µl) of the enzyme was used. The assay was done as described above except that the purified protein (0.3 µg protein in 10 µl buffer (0.1 M NaCl, 50 mM CH_3_COONa, pH 5.0)) was mixed with 90 µl of LAAO assay solution instead of 70 µl.

To determine enzyme kinetics, purified LAAO^WT^, LAAO^H223A^ and LAAO^R322A^ at 26.36 nM and LAAO^R90A^, LAAO^N172A^ and LAAO^Y372A^ at 158 nM were incubated with 2-fold serial dilutions of L-leucine from 10 to 1280 µM in an LAAO assay solution (0.1 M Tris-HCl pH 8.0, 2 mM OPD, 33.3 µg/ml peroxidase) in a 96-well ELISA plate (100 µl final assay volume). Samples were incubated at 37 °C for 1 h for LAAO^WT^, LAAO^H223A^ and LAAO^R322A^ or for 2 h for LAAO^R90A^, LAAO^N172A^ and LAAO^Y372A^ (mutants predicted to be inactive). The reaction was stopped by adding 50 µl of 0.5 M H_2_SO_4_, and the absorbance was determined at 490 nm as stated above. The initial velocity (µM per second) was defined as the change in the concentration of H_2_O_2_ produced by LAAO, which was plotted versus the concentration of substrate L-leucine before reaction (µM). The curves were then fitted using the nonlinear regression method in R software, from which the V_max_ and K_m_ (Michaelis constant) for each enzyme˗substrate reaction were derived. The k_cat_ (catalytic constant) was determined by dividing V_max_ by the enzyme concentration. The catalytic efficiency is defined as K_cat_/K_m_ (inverse µM per second).

### Determination of H_2_O_2_ production

To monitor the amount of H_2_O_2_ produced, 28 nM of LAAO^WT^ was added to DMEM phenol-free (Sigma, D1145) supplemented with 1% FCS and Glutamax and incubated at 37 °C, 5% CO_2_ for a period from zero to 6 h. Samples (50 µl) were collected into a 96-well ELISA plate (Thermo Fisher, 612U96) and mixed with 50 µl solution (4 mM OPD and 66.7 µg/ml peroxidase) for 5 min. The reaction was stopped by adding 50 µl of 0.5 M H_2_SO_4_. The absorbance was determined at 490 nm using a SpectraMax i3X and Pro 6.5 software. The amount of H_2_O_2_ content in the samples was calculated using a H_2_O_2_ standard curve.

### Cytotoxicity assay

Different cells show distinct sensitivity to LAAO toxicity [33]; thus, the Effective Concentration to reduce viability to 50% in 24 h (EC_50_) was calculated for each cell type (keratinocytes, HepG2 and SH-SY5Y) and batches of recombinant LAAO. LAAO cytotoxicity was tested using Alamar Blue cell viability reagent (Thermo Fisher, DAL1100) and the assay was performed as previously described [34] with modifications. After reaching 50–60% confluence, keratinocytes were treated with 2-fold serial dilutions from 7.5 to 0.00078125 µg/ml of freshly purified LAAO^WT^ in FAD standard medium containing 1% FCS for 24 h. The medium was then replaced with DMEM phenol-free (Gibco, 31053-028) containing 1% FCS, 4 mM L-glutamine and 10% Alamar Blue solution. Cells were continued to culture for 3 h before the fluorescence was read at 560 nm of excitation and 590 nm of emission in a POLARstar Galaxy fluorimeter using FLUOstar Galaxy software. For the comparison of cytotoxicity of LAAO^WT^ and mutants at the EC_50_ value of the WT, 14 nM of the WT and mutants were used to treat keratinocytes. For metabolic assays, normal keratinocytes were grown at a higher density, and thus a much higher EC_50_ was used in these experiments.

For the cytotoxicity assay of LAAO in SH-SY5Y or HepG2, cells were grown for 24 h before the treatment with LAAO^WT^ at 2-fold serial dilutions from 1.2 to 0.05 µg/ml and 7.0 to 0.5 µg/ml, respectively, for 24 h and processed as described above. Cytotoxicity at different conditions was calculated by comparing to the control set as 100% survival.

### Autophagosomes, organelles and oxidative stress measurements

After transfection with pEGFP-LC3 for 4 h, keratinocyte medium was refreshed with a fresh FAD standard medium containing 10% FCS. After 24 h post-transfection, the culture medium was changed to FAD standard medium containing 1% FCS and treated with LAAO^WT^ or mutant LAAO^R90A^ at 28 nM for 0.5, 1.5, 3 and 6 h and stained live with 100 nM Lysotracker red DND-99 (Thermo Fisher, L7528) for 1.5 h before ending the treatment. The probe is a red-fluorescent dye that is highly selective for labeling and tracking acidic organelles.

For mitochondrial membrane potential assay at 2 × EC_50_, cells were treated with 28 nM LAAO^WT^ for 0.5, 1.5, 3, and 6 h. To analyze the effect of LAAO^WT^ and catalytic dead mutant R90A on mitochondrial membrane potential, mitochondrial size and Feret’s diameter, ROS generation, 280 nM of LAAO^WT^ or LAAO^R90A^ was used for different time points. Cells were stained live with 100 nM MitoTracker Red CMXRos (Thermo Fisher, M7512), 5 µM CellRox Green Reagent (Thermo Fisher, C10444) and 1:10,000 Hoechst 33342 for 1.5 h. CellRox Green Reagent is a fluorogenic probe; the dye is weakly fluorescent while in a reduced state and exhibits bright green photostable fluorescence upon oxidation by reactive oxygen species and subsequent binding to DNA.

After live labeling with LysoTracker, MitoTracker Red CMXRos, or CellRox Green as described above, cells were washed once with PBS and fixed with 4% PFA (Alfa Aesar, J61899) for 10 min and stored in PBS at 4 °C when necessary. Images were captured at ×40 for 20 field of views/well of a 96-well plate.

### Monitoring autophagy flux

HepG2 cells stably expressing tandem mCherry-EGFP-LC3 were used to monitor autophagy flux [35] via a live-cell imaging-based approach. Cells were seeded in CellCarrier-384 ultra microplates (PerkinElmer, 6057300) at 10,000 cells/well/30 µl. After 16 h, the media of the culture was replaced with DMEM + GlutaMax supplemented with 10% FCS and 1:16,000 Hoechst 33342. Cells were left untreated or treated with 42 nM (2.4 µg/ml) LAAO^WT^ or LAAO^R90A^, 10 µM chloroquine in the presence or absence of 42 nM LAAO^WT^. Images were captured at ×40 in several fields of view, and the number of LC3 puncta under mCherry or EGFP channel was calculated, and the autophagy flux index was determined. The autophagy flux index (in each field of view) = total number of LC3 puncta in mCherry channel/total number of LC3 puncta in EGFP channel.

### Bioenergetic measurements

In order to measure glycolysis and mitochondrial respiration in real time, the Agilent Seahorse XFe96 Extracellular Flux Analyzer (Seahorse Biosciences) was used, as described previously [36]. The outputs were recorded as ECAR and OCR, respectively. In brief, keratinocytes were co-cultured with J2 in a 9 cm dish until they reached around 80% confluency. J2 cells were removed from the culture dish by briefly washing with Versene. Keratinocytes were seeded to a 1 μg/well collagen I (Sigma, C3867-1VL) precoated 96-well plate (Agilent, 102601-100) at a density of 19,000 cells/well/80 μl. The cells were rested at RT for 45 min before transferring to the incubator. After 16 h, the culture medium was changed to FAD standard medium containing 1% FCS and treated with 73 nM LAAO^WT^ or LAAO^R90A^ for 0.5, 1.5, 3.0, and 4.5 h. Culture media was then replaced with 175 μl of XF DMEM (Agilent, 103575-100) media containing 10 mM glucose, 1 mM sodium pyruvate, and 2 mM L-glutamine for Mito stress test or with 180 μl of the XF DMEM media containing only 2 mM L-glutamine for Glycolysis stress test. Cells were then incubated for 45 min at 37 °C without carbon dioxide to allow for cells to reach the ideal pH and temperature conditions required for the assay. The machine ran a 3-min mix and 3-min read cycle, which generated OCR and ECAR readings. During the assay, various compounds were injected via ports to see their effects on mitochondrial respiration and glycolysis. Three OCR measurements were recorded after each port injection, starting with oligomycin (1.5 μM), followed by FCCP (1 μM), and lastly a combination of rotenone and antimycin A (1 μM each), including Hoechst 33342 (1:10,000). ECAR measurements were recorded similarly except the port injection started with glucose (10 mM), followed by oligomycin (2 μM), and lastly 2DG (50 mM), including Hoechst 33342 (1:10,000). The final volume of each assay well became 250 μl. After analysis by the XFe96 Extracellular Flux Analyzer, the plate was imaged in the Opera Phenix (PerkinElmer) for capturing the image of the nucleus. Values of the 3rd reading were used for the calculation of mitochondrial activity and glycolytic activity parameters and calculated based on the Agilent Seahorse guidance.

### Detection of mitophagy using SH-SY5Y cells stably expressing mtKeima

SH-SY5Y stably expressing mtKeima cells were used to assess mitophagy activity induced by LAAO, hydrogen peroxide, and oligomycin in live cells, as the fluorescence of the Keima is visualized green (cytoplasmic mitochondria) or red (lysosomal mitochondria) at pH 7 or pH 4, respectively [37]. After 16 h of seeding cells, the media was replaced with DMEM supplemented with GlutaMax, 10% FCS and 1:16,000 Hoechst 33342. Cells were treated with 12.25 nM (0.7 µg/ml) LAAO^WT^, 12.25 nM LAAO^R90A^, 1 mM H_2_O_2_, or 10 µM oligomycin. Live images were captured at ×63 for 9 field of views/well of a 384-well plate at 0.1 h and every hour from 1 to 6 h. The images were analyzed using Columbus 2.8 (PerkinElmer), and the mitophagy index was determined. Briefly, cells and mtKeima were segmented, and the area of cytoplasmic mtKeima (green mtKeima; cytoplasmic mitochondria) and lysosomal mtKeima (red mtKeima; mitochondria in lysosomes) was determined. The mitophagy index (in each field of view) = total area of mitochondria in lysosomes/total area of cytoplasmic mitochondria.

### Immunocytochemistry and confocal microscopy image acquisition

After fixing with 4% PFA, keratinocytes were washed 3 times and permeabilized with 0.1% Triton X-100 and 10% FCS in PBS for 10 min and blocked with 50% FCS in PBS for 1 h at RT after 3 washings. Cells were incubated with anti-6x His-tagged antibody (DyLight 650) at 1:2000 (Abcam, ab117504) for 1 h or with anti-6x His-tagged antibody (Abcam, ab18184) at 1:1000 for 1 h, followed by Cy5 secondary antibody (Jackson Immuno Research) at 1:1000 for 1 h for the localization of LAAO. Cells were finally stained with 1:10,000 Hoechst 33342 (Thermo Fisher, H3570) for 10 min and were covered with 100 µl PBS. They were imaged at 20 images/well of a 96-well plate at ×40 (pixel size 0.1494 µm) using Opera Phenix High Content Screening System (PerkinElmer) with water immersion NA = 1.1.

Live images of cells were recorded in a humid chamber at 37 °C and 5% CO_2_ at ×40 (pixel size 0.1494 µm) or ×63 (pixel size 0.0949 µm) using Opera Phenix High Content Screening System (PerkinElmer). Excitation and emission wavelengths were adjusted based on manufacturer instructions.

### Image analysis

Image processing was done on a SuperServer 4048B-TR4FT high-performance server grade barebone computer equipped with 4 eight-core Intel Xeon E7-4809 v3 CPUs and 1 TB RAM running Ubuntu Linux 14.04 LTS. For desktop computation, a custom workstation was used with specifications Intel Core i9-7900X CPU and 128GB RAM memory running 64-bit Windows 10 Pro operating system.

Image processing was performed using Fiji/ImageJ [38] version 1.52n and Java 1.8.0_172. Images were acquired with Opera Phenix (PerkinElmer) and exported with Harmony 4.8 software (PerkinElmer) as single-channel 16-bit TIF images.

The number of nuclei was calculated by pre-processing the nuclear channel images with a 20-pixel radius mean filter followed by a watershed segmentation with a specified noise tolerance/prominence parameter. The nuclei area and ROS intensity were quantified by segmenting the nuclear channel images using a manually selected threshold and measuring both the area of the binary nuclear mask and the mean intensity of the underlying ROS channel pixels within the nuclear mask. The number of LC3 puncta was quantified by segmenting the LC3 images by watershed segmentation with suitably chosen noise tolerance/prominence parameter.

Lysosome images were pre-processed by contrast stretching and subsequently converted to 8-bit depth. Auto local segmentation was performed by Bernsen’s thresholding method (https://imagej.net/Auto_Local_Threshold) using parameters 5-pixel radius for local domain size and 30 for contrast threshold. Lysosome number and size (area) were measured on the segmented binary image using the Particle Analysis function after applying a size filter that kept only the particles larger than 0.05 µm^2^.

Mitochondria membrane potential was calculated as the mean intensity of the membrane potential channel pixels under the binary mask of tissue, which is calculated as follows. Images were pre-processed by contrast stretching followed by conversion to 8-bit depth. A mean filter with a 50-pixel radius was applied, and finally, a binary mask was generated by thresholding with the intensity value of 5.

Mitochondria were locally segmented by Bernsen’s thresholding method in a 5-pixel radius for local domain size and default value for contrast threshold. The mitochondrial number, area and Feret’s diameter, which represents the longest distance (μm) between any two points within a given mitochondrion. Values were quantified for each mitochondrial fragment with the size filter that only kept the particles larger than 0.05 µm^2^.

### Statistical analysis

All statistical tests were performed using GraphPad Prism 9.4, except where otherwise stated. For comparison of data from many groups, one-way analysis of variance (one-way ANOVA) was performed using Kruskal–Wallis and Dunn’s multiple comparison test to determine statistical significance. For experiments with time-dependent recorded from live imaging with multiple treatments, Mann–Whitney multiple comparisons (two-stage step-up Benjamini, Krieger, and Yekutieli) was performed as statistical test. The effective size between the two samples was calculated using unpaired *t*-test with Welch’s correction, a software built in GraphPad Prism 9.4 and shown as R-squared (eta squared). Data distributions were determined using Shapiro–Wilk normality test (Normality and Lognormality tests). Report of data distribution, effect size, and the number of images analyzed in each treatment per replicate/experiment are shown in Supplementary File S1.

## Results

### Insights into the structure of the LAAO catalytic site

**Fig. 1:**
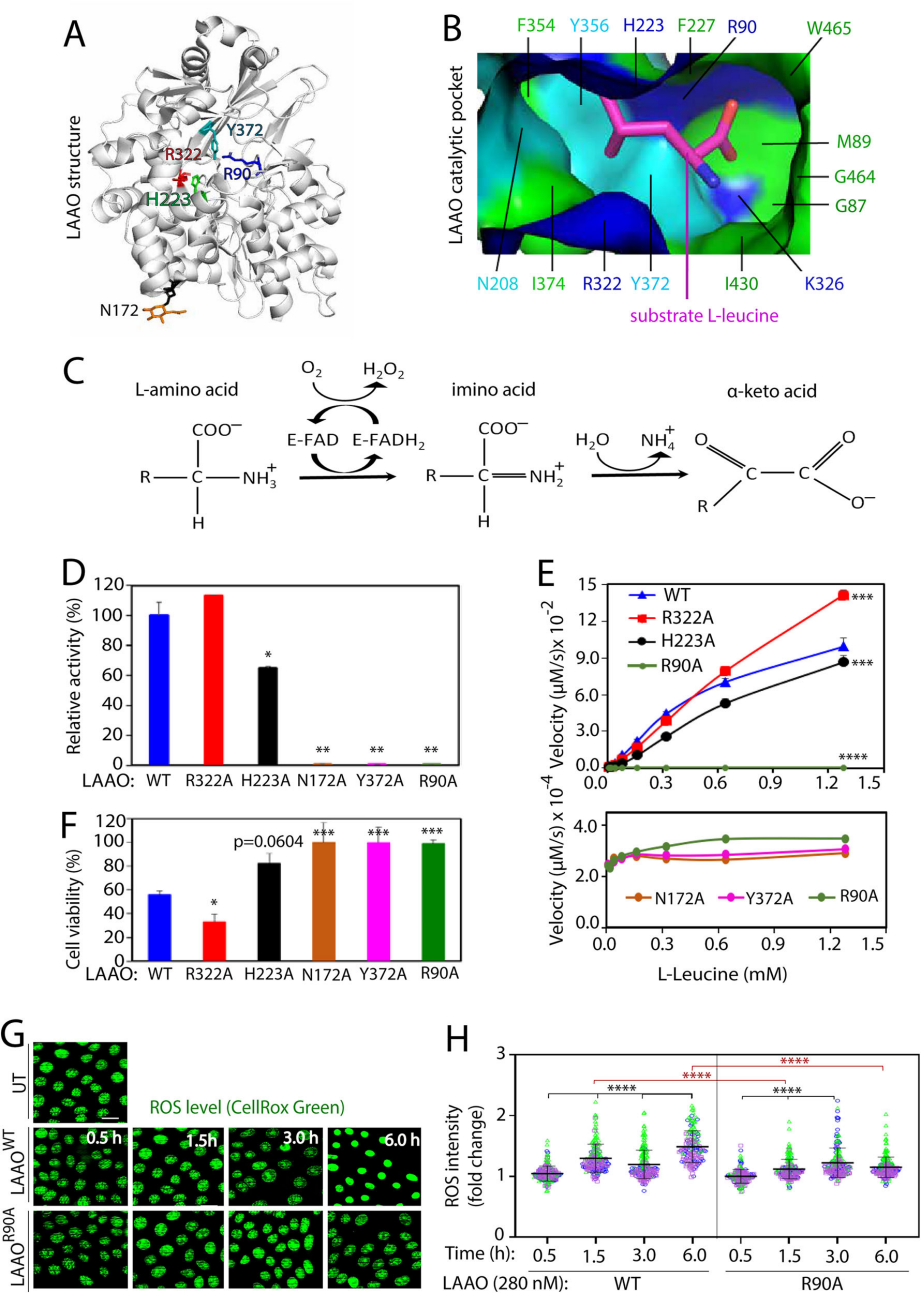
Catalytic activity, cell cytotoxicity, and oxidative stress of recombinant *B. atrox* LAAO^WT^ and mutants. **A**, **B** 3D structure of LAAO and its catalytic site. **A** Monomeric structure of *B. atrox* LAAO showing the predicted catalytic and substrate binding residues as sticks: Y372, R322, R90, H223, N172. **B** Surface of the substrate binding pocket of LAAO (*A. H. pallas* venom (PDB 1TDN) showing positively charged amino acid residues (blue), polar residues (cyan), and hydrophobic residues (green). L-leucine substrate is shown as a magenta stick. **C** L-amino acid oxidation reaction catalyzed by LAAO^WT^ with cofactor FAD (E-FAD) generating H_2_O_2_ and ammonia as byproducts. **D**, **E** Biochemical characterization of LAAO mutants. Activity of purified LAAO^WT^ or LAAO mutants (52.72 nM) compared to wild type (**D**, arbitrarily set at 100) or their initial velocity V (**E**; µM per second, Y axis) using nonlinear regression method in R software. The initial velocity V is defined as the change in the concentration of H_2_O_2_ produced by LAAO at each concentration of substrate L-leucine (mM, X axis). In the bottom panel, catalytic activities of selected mutants are shown magnified (×100). **F** Keratinocyte cell viability was assessed by incubation 14 nM of LAAO^WT^ or mutants. **G**, **H** Mutated LAAO generates significantly less cellular reactive oxygen species (ROS) than LAAO^WT^. Keratinocytes were treated with vehicle, 280 nM LAAO^WT^ or LAAO^R90A^, live-stained with CellRox Green (green) and fixed before confocal imaging. Representative images (**G**) and quantification (**H**) are shown; samples from independent replicates are shown in blue, green, or pink colors. All assays were done as multiple technical replicates in three independent biological replicates (thereafter *N* = 3) and data shown as mean, and error bars represent standard deviation. Statistical analysis was performed as unpaired *t*-test (**D**–**F**) or One-way ANOVA and Kruskal–Wallis and Dunn’s multiple comparison test (**H**). ****P ≤ 0.0001, ***P ≤ 0.001, **P ≤ 0.01, *P ≤ 0.05. In (**H**), black asterisks represent comparison within a single treatment group; red asterisk compares across different treatments. Number of all images used for the analysis, data distribution and effective size eta squared (ƞ^2^) are reported in File S1. Scale bar: 20 µm.

Snake venom LAAOs have very similar amino acid sequence (Fig. S1) and structure [15]. *B. atrox* venom LAAO monomeric structure (Fig. 1A) is most similar to that of *Agkistrodon halys pallas* (*A. H. pallas*) venom LAAO (RMSD 0.38 Å using PyMOL analysis). Here, we investigated in depth several amino acid residues previously proposed to form the LAAO catalytic pocket or that have a close interaction with substrate and cofactor FAD (Fig. 1B) [15, 39]. Arginine residue (R90) has been suggested to interact with the cofactor flavin-adenine dinucleotide (FAD) and the substrate [15], while tyrosine at position 372 (Y372) is proposed as a substrate/ligand binding residue [39]. A glycosylated site at an asparagine residue (N172) seems important for catalysis (Fig. 1A), albeit a surface residue [15]. Finally, R322 and H223 have been proposed as important residues in the catalytic pocket [39]. We successfully produced various recombinant proteins mutated in these residues and analyzed their catalytic efficiency in the oxidation reaction (Fig. 1C) [9] and *in cellulo* effects (see below).

### *B. atrox* LAAO catalytic kinetics reveals the essential role of residues R90, Y372 and N172

The production of recombinant active LAAO has its challenges due to its high toxicity, insolubility in bacteria, distinct glycosylation by yeast [30, 40, 41] or limited secretion in mammalian systems [12, 30]. Using HEK293T cells, we successfully obtained recombinant LAAO and novel mutated versions of the protein on the proposed residues for LAAO catalyses (R322, H223, N172, Y372 and R90; Fig. S2A).

Among the preferred in vitro substrates for native *B. atrox* LAAO (L-Tyr, L-Phe, L-Ala, and L-Leu) [13], we chose L-Leu to determine the in vitro catalytic activity of LAAO recombinant proteins. The mutants LAAO^R90A^, LAAO^N172A^ and LAAO^Y372A^ showed a strong reduction in their activity (Fig. 1D), while LAAO^H223A^ showed partial inhibition of catalysis (Fig. 1D) as previously reported [41]. Unexpectedly, LAAO^R322A^ had a small but reproducible activity when compared to LAAO^WT^. Catalytic kinetics (Fig. 1E) showed that the enzymatic velocity of LAAO^R322A^ (at 26.36 nM) is slower than the LAAO^WT^ (L-Leu concentration at ≤300 µM) but became faster at substrate concentrations above 600 µM. The catalytic efficiency (K_cat_/K_m_) of recombinant LAAO^WT^ and the relative catalytic activity were strongly reduced for all mutants, apart from LAAO^R322A^ (Table 1). The contribution of residue R322 to catalysis remains unclear, and it will be investigated elsewhere. It is feasible that R322 may cooperate with other residues to influence catalyses, as shown with equivalent mutations in recombinant LAAO from *Naja naja* snake [12]. Our data on *B. atrox* LAAO catalyses is innovative as (i) they strengthen the case for the essential roles of R90, Y372 and N172, but not R322, and (ii) identify new inactive mutants to perform mechanistic experiments.

**Table 1:** Enzymatic kinetics of LAAO^WT^ and its mutants.

Catalytic kinetics						
LAAO	WT	R322A	H223A	R90A	N172A	Y372A
Kcat (1/s)	4.53 ± 0.32	17.4 ± 0.91	7.28 ± 1.62	ND	ND	ND
Km (µM)	501 ± 46	2410 ± 142	1457 ± 392	ND	ND	ND
Kcat/Km (1/µM.s)	9.08E-03	*	5.50E-03	ND	ND	ND
LAAO relative activity-high L-Leucine concentration						
LAAO	WT	R322A	H223A	R90A	N172A	Y372A
Activity	1	1.13 ± 0.005	0.64 ± 0.011	0.0044	0.0041	0.0028

The results were derived from Fig. 1E and curves fitting using the nonlinear regression method in R software. Results are displayed as mean ± standard deviation. *N* = 3.

ND, not detected.

*LAAO^R322A^ catalytic activity depends on the substrate concentration, as shown in the bottom rows.

Consistent with native LAAO [18], recombinant LAAO^WT^ was toxic to normal keratinocytes (Effective Concentration (EC_50_) of 0.8 µg/ml or 14 nM; Fig. S2B). Viability assays using LAAO mutants at the same concentration (14 nM; Fig. 1F) demonstrated that LAAO^R322A^ potently reduced viability to a greater extent than LAAO^WT^, while a small reduction in cell viability was promoted by LAAO^H223A^ (Fig. 1F). The mutants N172A, Y372A or R90A did not interfere with the metabolic fitness of normal keratinocytes.

As shown previously in keratinocytes [18], hydrogen peroxide is a toxic byproduct of LAAO oxidation (Fig.1C) and can be converted to reactive oxygen species (ROS). We found that in cell culture media, recombinant LAAO^WT^ (28 nM) exponentially produces H_2_O_2_ to 10 mM in 6 h (Fig. S2C). To detect ROS after LAAO treatment (20 × EC_50_), cells were stained with CellRox Green, a cell-permeable dye (Fig. 1G, H). ROS levels fluctuated but were highest (increased by 49.1%) after 6 h incubation with LAAO^WT^. A small, transient burst of ROS levels (increased by 19.7%) was seen at 3 h after LAAO^R90A^ incubation (timing and intensity distinct from LAAO^WT^). Thus, similar to native LAAO [42], recombinant *B. atrox* LAAO^WT^ elevates cellular oxidative stress in a catalysis-dependent manner (this work).

### LAAO disrupts autophagy flux and lysosome function

**Fig. 2:**
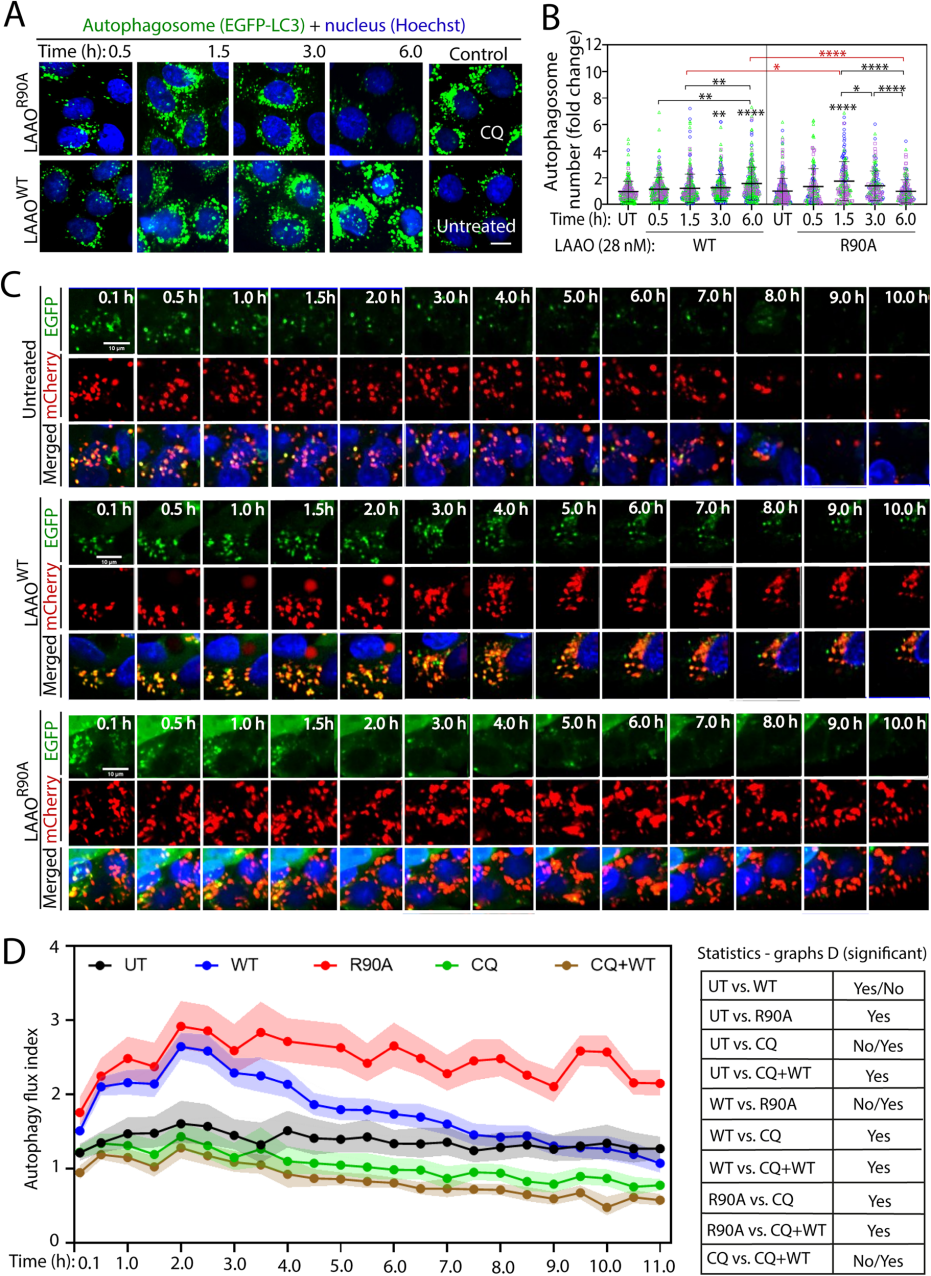
Autophagic response of keratinocytes and HepG2 cells in the presence of LAAO^WT^ or LAAO^R90A^. **A**, **B** Mutated LAAO^R90A^ (catalytic dead) transiently stimulate autophagy, while LAAO^WT^ promotes stronger and sustained stimulation. Keratinocytes were transfected with pGEF-LC3 as a marker for autophagosomes and after 24 h, they were treated with 28 nM of LAAO^WT^ or LAAO^R90A^ for 6 h. Controls are untreated cells or those treated with 10 µM chloroquine (CQ). Representative confocal images of GFP tag and nucleus (Hoechst) (**A**) and quantification (**B**) of the number of LC3 puncta in each sample is normalized to untreated cells. Bars indicate mean and error bars show standard deviation. Three independent biological replicates (*N* = 3) are represented in different colors (blue, green, or pink). Multiple statistical comparisons among different groups were performed using One-way ANOVA and Kruskal–Wallis and Dunn’s multiple comparison test. **C**, **D** Autophagy flux is impaired by exposure to LAAO^WT^. HepG2 cells stably expressing tandem mCherry-EGFP-LC3 were left untreated or incubated with either LAAO^WT^ or LAAO^R90A^ (1 × EC_50_, 42 nM for HepG2 cells). **C** Representative confocal live imaging of HepG2 cells, images were captured every 30 min. For each sample, images show the fluorescence of EGFP-LC3 (top row) or mCherry-LC3 (middle row) and the merged image (bottom row) with nuclei stained with Hoechst 33342 (blue). Representative images of controls are shown in Fig. S3: chloroquine (CQ) or chloroquine + LAAO^WT^ (CQ + WT). **D** Fusion of autophagosomes with lysosomes is prevented by LAAO toxicity. Autophagy flux index is the ratio between the number of autophagolysosomes (red LC3 puncta in acidic compartments such as lysosomes) and the number of autophagosomes prior to that (green LC3 puncta, acidic environment quenches green fluorescent). Time-series analyses are shown as geometric mean with error, 95% confidence interval (95% CI) added as light-colored shadow (*N* = 3). Table on the right shows the overall comparison. Statistics and the detail of comparisons of all samples in all time points are shown in Supplementary File S1. Multiple statistical comparisons among different groups were performed using Mann–Whitney multiple comparisons (two-stage step-up (Benjamini, Krieger, and Yekutieli)) (**D**). Black asterisks show comparison within a single treatment group; red asterisks show comparison across different treatments. ****P ≤ 0.0001, **P ≤ 0.01, *P ≤ 0.05. Number of all images used for the analysis, data distribution and effective size eta squared (ƞ^2^) are reported in File S1. Scale bar: 10 µm.

Autophagy is the earliest detectable cell stress response caused by LAAO treatment of primary cells [18]. However, how envenomation triggers or modulates autophagic responses is not known. To investigate further LAAO-induced autophagy, we expressed EGFP-LC3 as a marker for autophagosomes in keratinocytes. The number of LC3 puncta (i.e., as a proxy for autophagosome numbers) at any given time is a balance between autophagosome de novo formation and degradation. When compared to untreated controls and consistent with native LAAO [18], LAAO^WT^ incubation significantly increased the number of LC3 puncta by 27.9% and 47.1% after 3 and 6 h, respectively (Fig. 2A, B). In contrast, the catalytically dead mutant (LAAO^R90A^) triggered a transient surge in the number of LC3 puncta by 72.6% at 1.5 h, suggesting that autophagosomes were induced and quickly degraded at later time points, returning to basal levels (Fig. 2A, B). As the number of LC3 puncta remains high after LAAO^WT^ treatment, we surmise that autophagy flux is promoted, but autophagosome degradation in lysosomes might be impaired.

To differentiate between autophagosome initiation and degradation, autophagy flux analysis was performed using a different, well-established model, Hep2G2 cells expressing mCherry-EGFP-LC3 (Fig. 2D) [35]. Autophagy flux index is calculated as the ratio (mCherry/GFP) in LC3 puncta. It measures the rate at which autophagosomes reach the lysosomal compartment, via quenching of EGFP fluorescence (sensitive to low pH in lysosomes) while mCherry fluorescence is not affected [35]. Basal autophagy flux index of untreated cells showed the disappearance of EGFP fluorescence in LC3 puncta as autophagosomes fuse with the acidic compartment of lysosomes (Fig. 2C). Treatment with chloroquine to impair lysosome acidification and content degradation [43] reduced the autophagy flux index throughout the time course (Fig. 2D, S3A).

Autophagy flux index was increased in LAAO^WT^- and LAAO^R90A^-treated for up to 2 h (EC_50_ = 42 nM; Fig. 2D) [33], indicating higher degradation of autophagosomes with either treatment. However, after 3 h, the autophagy flux index in LAAO^WT^ samples decreased, as clearance of autophagosomes in lysosomes was reduced, returning to basal levels with time (Fig. 2C, D). In contrast, inactive LAAO^R90A^ treatment induces a higher autophagy flux index and is significantly distinct from untreated cells (Fig. 2C, D). The data suggest that the inactive LAAO mutant continues to generate autophagosome formation and effective degradation in HepG2 cells (Fig. 2D). Such continuous induction of autophagosomes by inactive LAAO treatment may reflect a catalysis-independent phenotype of clearing an extraneous exogenous protein. Finally, the higher autophagy flux caused by LAAO^WT^ treatment is blocked by chloroquine treatment (Fig. 2D, S3B), confirming that the observed increase in autophagy flux index up to 3 h requires a functional lysosome. We concluded that the catalytic activity of LAAO inhibits lysosome function and impairs autophagosome degradation.

Thus, LAAO^R90A^ phenotype of sustained high autophagy flux index in HepG2 cells (Fig. 2C, D) is consistent with the data on keratinocytes (Fig. 2B) showing a transient increase of LC3 puncta likely from a fast clearance in lysosomes. Whereas the interpretation of inhibition of autophagosome clearance by LAAO^WT^ is consistent across HepG2 and keratinocyte models, it is possible that cell-specific differences may be present, due to distinct survival abilities of immortalized cell lines (HepG2) versus normal cells (keratinocytes). Overall, this set of results suggests that LAAO activity may inhibit autophagy flux either by preventing lysosomal acidification or fusion of autophagosomes with lysosomes.

**Fig. 3:**
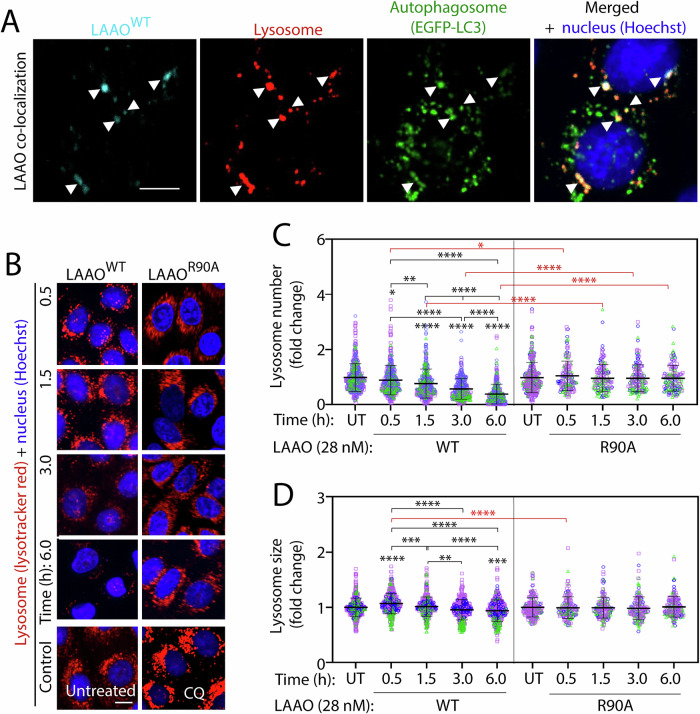
*B. atrox* LAAO internalization and lysosomal responses. **A** LAAO^WT^ partially colocalises with lysosomes and autophagosomes. Confocal images of keratinocytes transiently transfected with pEGFP-LC3 and treated with 28 nM LAAO^WT^ for 3 h. White arrowheads indicate co-localization of LAAO^WT^ (cyan) with LC3 (green) and lysosome (red). **B**–**D** Lysosome size and numbers are reduced following exposure to LAAO^WT^. Keratinocytes were live-stained for lysosomes (100 nM Lysotracker red DND-99) or nucleus (1:10,000 Hoechst) for 1.5 h. Cells were then incubated with 28 nM of LAAO^WT^ or catalytic dead LAAO^R90A^ or 10 µM chloroquine (CQ) for up to 6 h. Confocal live images (**B**) were quantified to obtain lysosome numbers (**C**) and sizes (**D**). Values are expressed as fold change relative to untreated samples. Bars indicate the mean, and error bars show the standard deviation. Multiple statistical comparisons among different groups were performed using one-way ANOVA and Kruskal–Wallis and Dunn’s multiple comparison test, *N* = 3; independent biological replicates are represented in different colors (blue, green, or pink). Black asterisks compare within a single treatment group; red asterisks compare across different treatments. ****P ≤ 0.0001, ***P ≤ 0.001, **P ≤ 0.01, *P ≤ 0.05. Number of all images used for the analysis, data distribution and effective size eta squared (ƞ^2^) are reported in File S1. Scale bar: 10 µm.

Next, we investigated further the intracellular localization of LAAO [18]. In keratinocytes, recombinant His-tagged LAAO^WT^ was internalized and found in vesicles, where it partially co-localized with autophagosomes (pEGFP-LC3) and lysosomes (Lysotracker red DND-99; Fig. 3A). A progressive reduction of lysosome number (66%) and size (6.4%) was induced by treatment with wild-type (6 h), but not the catalytic dead LAAO (Fig. 3B–D). Taken together, we demonstrate that recombinant LAAO accumulates in autophagosomes and alters lysosome number and morphology in a catalysis-dependent manner. We surmise that the lysosome dysfunction triggered by LAAO would delay or inhibit autophagosome degradation and autophagy flux, therefore exacerbating cytotoxicity.

### LAAO promotes mitochondria dysfunction and morphological changes

**Fig. 4:**
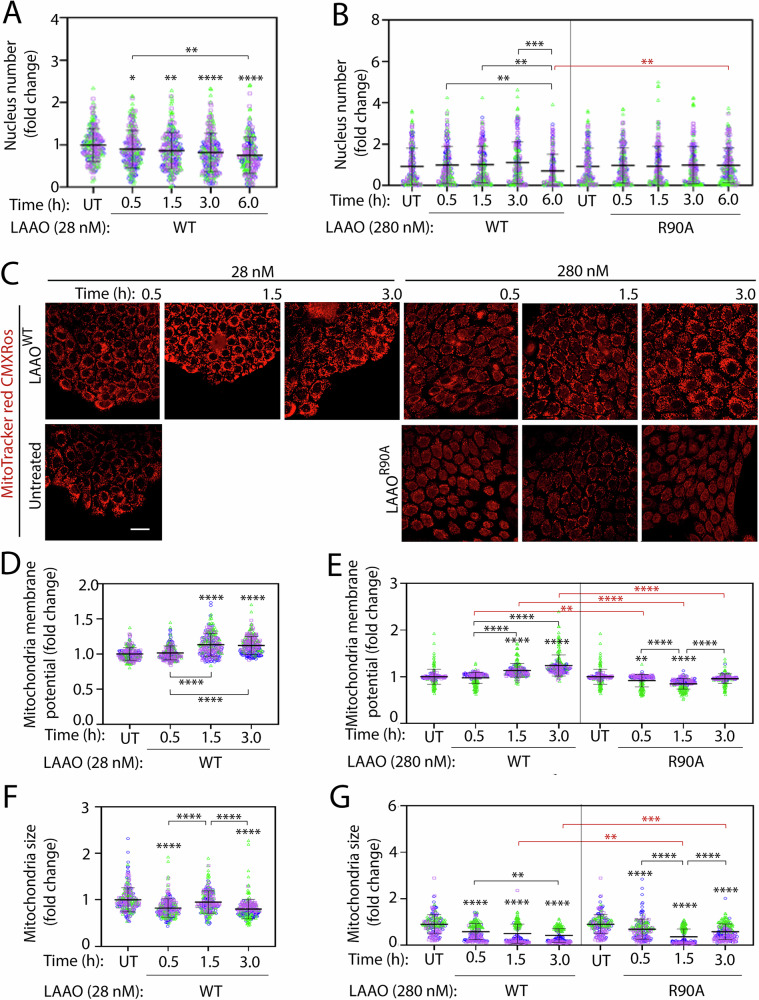
LAAO treatment reduces cell number and mitochondria size and function. Normal keratinocytes were treated with recombinant LAAO proteins at different concentrations (28 nM or 280 nM) for up to 6 h. Cells were stained live with Hoechst to count cell numbers and MitoTracker Red CMRos to show mitochondria membrane potential. Cells were fixed before confocal image acquisition. **A**, **B** Reduction of nucleus number following exposure to LAAO^WT^. Cells were stained with 1:10,000 Hoechst and imaged at 20 field of views/well regardless of the number of nuclei presence in the well. The total number of nuclei was quantified and expressed relative to untreated controls. **C**–**E** Mitochondria membrane potential is increased by LAAO^WT^ treatment. **C** Representative images of keratinocytes stained with MitoTracker CMXRos. **D**, **E** Quantification of mitochondria membrane potential. **F**, **G** LAAO^WT^ promotes mitochondria fragmentation. Graphs show quantification of Mitochondrial size following different treatments. All graphs show values normalized to the untreated samples. Bars indicate the mean, and error bars show the standard deviation. Multiple statistical comparisons among different groups were performed using one-way ANOVA and Kruskal–Wallis and Dunn’s multiple comparison test, *N* = 3; independent biological replicates and are represented in blue, green, or pink colors. Black asterisks compare within a single treatment group; red asterisks compare across different treatments. ****P ≤ 0.0001, ***P ≤ 0.001, **P ≤ 0.01. Number of all images used for the analysis, data distribution and effective size eta squared (ƞ^2^) are reported in File S1. Scale bar: 20 µm.

As the amount of LAAO in venoms from different snake species can vary extensively [44], higher concentrations of LAAO^WT^ (280 nM) were tested in parallel to reflect the variability of LAAO concentration in different venoms. The early alterations on autophagy flux and lysosomal dynamics caused by LAAO^WT^ were not accompanied by extensive cell detachment as quantified by the nuclei number as a proxy (Fig. 4A). A significant and time-dependent decrease in cell number was most evident at 6 h (26.7%; Fig. 4A, B). Higher LAAO concentration promoted cell removal (39.8%) after 6 h incubation, while the catalytically dead LAAO^R90A^ did not (Fig. 4B). Altogether, our results indicate that cell detachment induced by wild-type LAAO does not temporally correlate with the changes in autophagy flux and lysosomal function breakdown observed at earlier time points.

Using MitoTracker Red CMXRos (Fig. 4C–G), we found that mitochondria function was perturbed in two different ways. First, mitochondrial membrane potential was altered after treatment with LAAO^WT^ (1.5 h onward; Fig. 4C–E). A small, but significant increase in MitoTracker Red intensity at 1.5 h and 3 h was observed upon incubation with LAAO^WT^ (11.4% and 20.5%, respectively; Fig. 4D, E), but not LAAO^R90A^. Second, the membrane potential disruption by LAAO^WT^ was accompanied by a strong reduction in mitochondria size at 3 h (23.4%; Fig. 4F and 61.1%; Fig. 4G). At higher concentrations, catalytically inactive LAAO^R90A^ also promoted a decline in mitochondria size, but less intensely than wild-type LAAO, except for an early, acute decrease in mitochondria size at 1.5 h (60%; Fig. 4G).

**Fig. 5:**
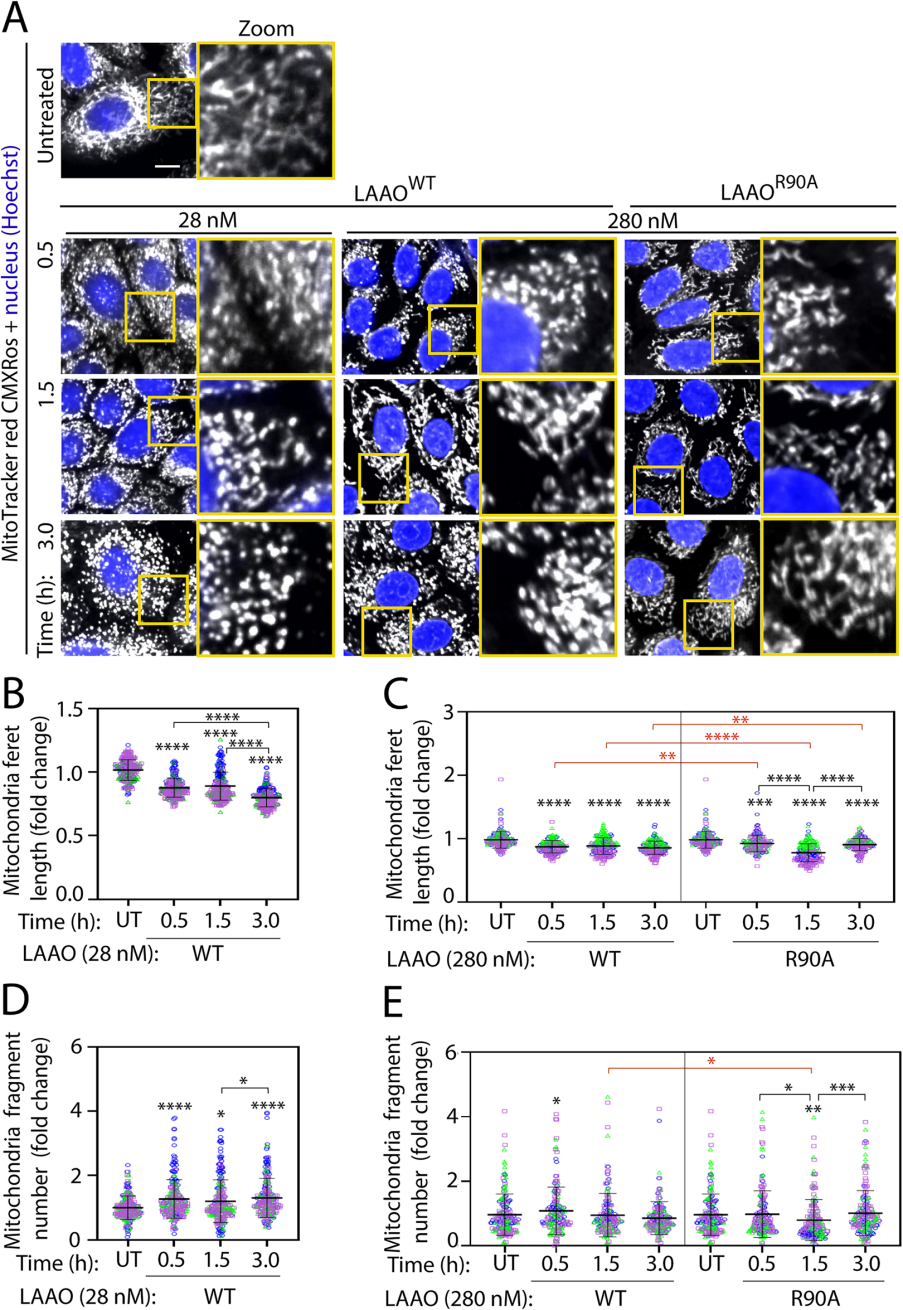
*B. atrox* LAAO promotes mitochondria fragmentation. Normal keratinocytes were treated with recombinant LAAO proteins at different concentrations (28 nM or 280 nM) for up to 3 h. **A** Mitochondria morphology changes induced by LAAO^WT^. Cells were stained live with Hoeschst and MitoTracker CMXRos and fixed before confocal microscopy imaging. Representative images show Mitochondria and nuclei are shown in gray and blue, respectively. Yellow box shows the region of interest that is enlarged on the right-side panel. **B**–**E** Quantification of mitochondria morphology. Images were processed to quantify mitochondria Ferret diameter (**B**, **C**) or number of mitochondria fragments (**D**, **E**). Mitochondria Feret’s diameter (i.e., the longest distance (μm) between any two points within a given mitochondrion. All values are normalized to the untreated (UT) samples and expressed as fold change. Bars indicate the mean, and error bars show the standard deviation. Multiple statistical comparisons among different groups were performed using one-way ANOVA and Kruskal–Wallis and Dunn’s multiple comparison test. *N* = 3; independent biological replicates are represented in blue, green, or pink colors. Black asterisks compare within a single treatment group; red asterisks compare across different treatments. ****P ≤ 0.0001, ***P ≤ 0.001, **P ≤ 0.01, *P ≤ 0.05. Number of all images used for the analysis, data distribution and effective size eta squared (ƞ^2^) are reported in File S1. Scale bar: 10 µm.

Consistent with the above data, mitochondria Feret’s length (Fig. 5A–C) and mitochondria fragment number (Fig. 5D, E) were consistent with the measured mitochondria size (Fig. 4F, G), i.e., bigger mitochondria size – longer ferret length – smaller mitochondria fragment number or smaller mitochondria size – shorter ferret length – larger mitochondria fragment number except the treatment at higher concentration with LAAO^WT^ at 3 h and LAAO^R90A^ at 1.5 h which resulted in smaller mitochondria size (38.8% and 26.6%) – shorter ferret length (85.3% and 75.0%) – smaller mitochondria fragment number (85.4% and 75.6%), suggesting mitochondria lost. The increase in mitochondria size (54.7%) – Feret’s length (92.8%) – mitochondria fragment number (100.0%) at 3 h from 1.5 h when treated with LAAO^R90A^ indicated mitochondria recovery when treated with inactive LAAO. The reasons why inactive LAAO altered mitochondria morphology transiently in the absence of higher oxidative stress are currently unclear.

The discrepancy between the timing of mitochondrial fragmentation with different LAAO concentrations (Fig. 5D, E) may be due to the ability of a higher dosage of LAAO^WT^ to fragment mitochondria more potently. The phenotype with 280 nM LAAO^WT^ could result from (i) a faster and more efficient fragmentation, which tails off to steady state by 1.5 h, and (ii) smaller mitochondria fragments are more readily engulfed by autophagosomes and cleared [45]. The net result was no significant increase in fragment numbers. We surmise that the high oxidative stress induced by LAAO catalysis increases mitochondria fragmentation and reduces their size and Feret’s diameter.

### Cell metabolism and energy production are strongly disturbed by LAAO

**Fig. 6:**
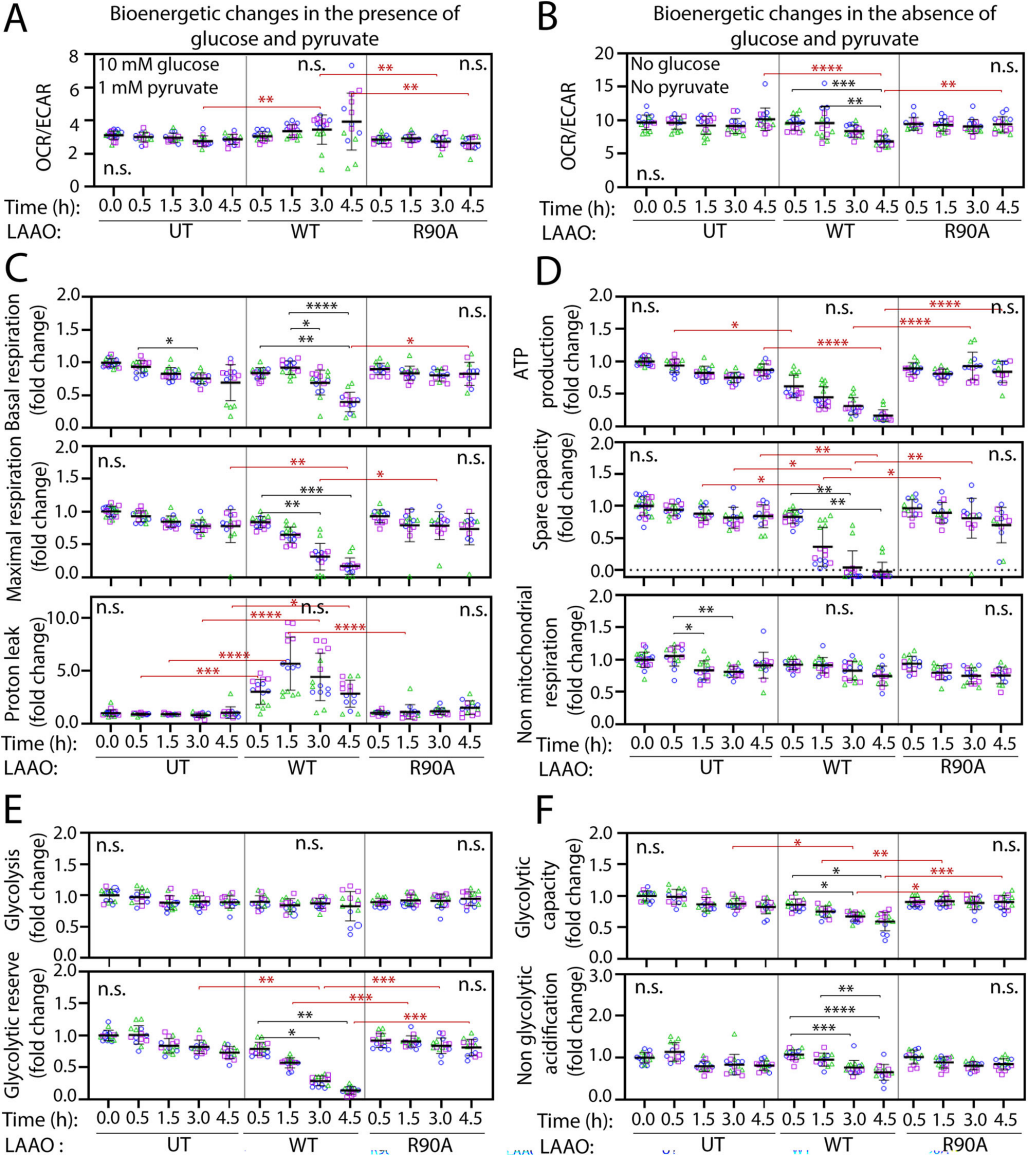
Mitochondrial dysfunction and metabolic stress are promoted by LAAO catalysis. Oxygen consumption rate (OCR) and extracellular acidification rate (ECAR) were measured in keratinocytes untreated (UT) or treated with 2 × EC_50_ (73 nM for SeaHorse experiments) of LAAO^WT^ or LAAO^R90A^ for up to 4.5 h as described in “Methods.” Recorded OCR and ECAR profiles for Cell Mito Stress and Glycolysis Stress tests are shown in Fig. S4. **A**, **B** Measurements of bioenergetic changes in the presence (**A**) or absence (**B**) of glucose and pyruvate. Values are obtained by calculating OCR/ECAR ratio at basal respiration. **C**, **D** Respiration and ATP production are severely impaired by LAAO catalyses. Metabolic parameters are calculated from the Mitochondrial Stress test: non-mitochondrial and mitochondrial respiration (basal and maximal), proton leak, ATP production and spare capacity. **E**, **F** Glycolytic reserve and capacity are reduced by LAAO^WT^ treatment. Glycolytic parameters were calculated from the Glycolysis Stress test: glycolysis, non-glycolytic acidification, glycolytic reserve, and capacity. Bars indicate the mean, and error bars show the standard deviation. Multiple statistical comparisons among different groups were performed using one-way ANOVA and Kruskal–Wallis and Dunn’s multiple comparison test, *N* = 3; independent biological replicates are represented in blue, green, or pink colors. Black asterisks compare within a single treatment group; red asterisks compare across different treatments. ****P ≤ 0.0001, ***P ≤ 0.001, **P ≤ 0.01. Number of all images used for the analysis, data distribution and effective size eta squared (ƞ^2^) are reported in File S1.

The above morphometric changes suggest that mitochondrial function is severely impaired by LAAO. As mitochondria have an essential role in generating cellular energy, we carried out bioenergetic measurements (see “Methods” and figure legends). Following a time course, the profiles for Oxygen Consumption Rate (OCR) and Extracellular Acidification Rate (ECAR) were recorded in the presence or absence of recombinant LAAO proteins (Fig. S4). Normal keratinocytes have a preference for glycolysis [46], but switched to oxidative phosphorylation in glucose- and pyruvate-deprived conditions (Fig. 6A, B). LAAO^WT^ treatment impaired the ability to adapt energetically in the absence of glucose or pyruvate, while LAAO^R90A^ had no effect (Fig. 6A, B).

In contrast to controls or inactive LAAO, both basal and maximal respiration levels were severely reduced by LAAO^WT^ treatment at 4.5 h (60.0% and 85%, respectively) and correlated with high levels of proton leakage (Fig. 6C). During the time course investigated, there were fluctuations in ATP production and spare capacity in controls or catalytic inactive LAAO, but these were not significant. In contrast, both ATP generation and spare capacity were strongly decreased by LAAO^WT^ incubation at 4.5 h (86.8%; Fig. 6D). However, non-mitochondria respiration (Fig. 6D) and glycolysis (Fig. 6E) were not altered by either treatment. Instead, a significant, strong decline in glycolytic reserve levels (86.9%), and milder reductions in glycolytic capacity (45%) and non-glycolytic acidification (40.0%) were induced by LAAO^WT^ when compared to non-treated controls (time zero, Fig. 6E, F, S4).

Taken together, our data indicate that LAAO interferes with energy production and impairs the switch to alternative energy sources in a catalysis-dependent manner. The outcome is a significant reduction in respiration, glycolytic reserve and capacity and a shutdown of ATP production.

### LAAO induces mitophagy

**Fig. 7:**
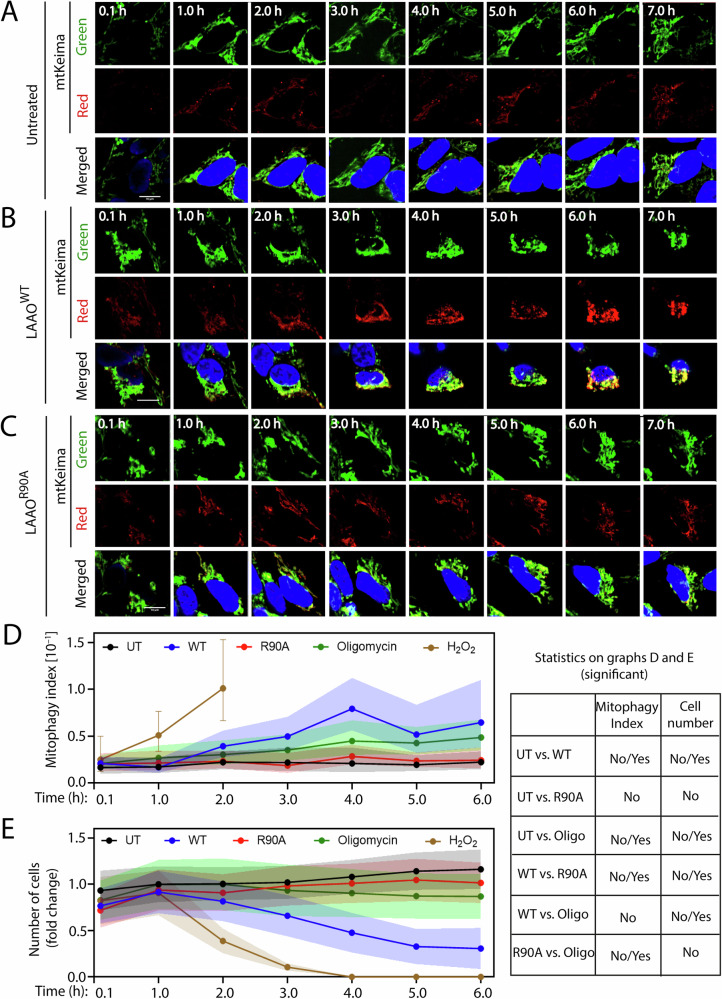
LAAO cytotoxicity triggers mitophagy, but not clearance of mitochondria. SH-SY5Y cells stably expressing mtKeima (mitochondria-targeted dual fluorescent protein) untreated (**A**) or following treatment with **B** LAAO^WT^ or **C** LAAO^R90A^ (2.8 × EC_50_ or 12.25 nM for SH-SY5Y cells) and imaged live for up to 7 h using confocal microscopy. **A**–**C** Mitophagy dynamics is prevented by LAAO treatment. Representative frames at different time points (columns) showing fluorescence of cytoplasmic mtKeima green (top row), lysosomal mtKeima red (middle row), and the merged images (bottom row), including nuclei stained with Hoechst 33342 (at the lower concentration used, the nucleus cannot be fully stained at 0.1 h). As controls, cells were also treated with 10 µM oligomycin or 1 mM H_2_O_2_ (shown in Fig. S5). **D** LAAO catalysis prevents mitochondria degradation in lysosomes. Mitophagy index assesses the change of mitochondria state from cytoplasmic mtKeima (green at pH 7.0) to a lysosomal mtKeima (red at pH 4.0). Mitophagy index was calculated (mean area of red mtKeima/mean area of green mtKeima) per image and normalized to values of untreated (UT) samples. **E** LAAO treatment reduces cell number at later time points. Number of cells present after treatment for up to 6 h. Treatment with 1 mM H_2_O_2_ was toxic to SH-SYS5 cells and led to substantial cell detachment after 2–3 h. Values in (**D**, **E**) are shown as geometric mean with error, 95% confidence interval (95% CI) added as colored shade or bar for H_2_O_2_. Multiple statistical comparisons among different groups were performed using Mann–Whitney multiple comparisons (two-stage step-up (Benjamini, Krieger, and Yekutieli). *N* = 3. ****P ≤ 0.0001, ***P ≤ 0.001. Table on the right shows the *P*-value for overall comparison. Number of all images used for the analysis, data distribution and effective size eta squared (ƞ^2^) are reported in File S1. Scale bar: 10 µm.

The severe impairment of energy production and mitochondria morphology suggests that defective mitochondria could be cleared by mitophagy. However, we postulated that mitophagy may also be compromised, as the strong reduction in lysosome numbers and size may prevent autophagosome degradation (Fig. 3B–D). We evaluated the mitophagy index using live imaging and SH-SY5Y cells stably expressing mt-Keima (Fig. 7), a pH-sensitive fluorescent protein [37] that is targeted to the mitochondria. During mitophagy, in the acidic lysosomal environment, mt-Keima fluorescence emission shifts from the shorter (green) to longer wavelength (red). The mitophagy index was calculated as a ratio between the segmented area of the two mt-Keima in each image (lysosomal and cytoplasmic). As a positive control, hydrogen peroxide treatment to induce oxidative stress led to a progressive co-localization of green and red mt-Keima (Fig. S5A). In contrast, addition of oligomycin to inactivate mammalian F_0_F_1_-ATPsynthase did not promote mt-Keima shift to red fluorescence, consistent with the prevention of H^+^ ion transport and lysosome acidification (Fig. S5B).

SH-SY5Y mt-Keima EC_50_ was determined in the presence of recombinant LAAO^WT^ (EC_50_ = 4.38 nM). Basal levels of mitophagy were similar in untreated samples and after incubation with inactive LAAO (LAAO^R90A^, Fig. 7A, C). LAAO^WT^ treatment promoted a persistent conversion to red emission from 2 h onward (Fig. 7B): the mitophagy index baseline of 0.16 was increased to 0.79, a maximum at 4 h (Fig. 7D) and was significantly distinct from its inactive counterpart. There were no significant differences in mitophagy index over time between cells treated with LAAO^R90A^ or controls (Fig. 7D). Mitochondria degradation was not observed during the time course, due to the high sensitivity of SH-SY5Y cells to LAAO treatment.

As controls, no significant changes in cell numbers were observed in untreated, oligomycin- or inactive LAAO-treated samples (Fig. 7E). Upon treatment with H_2_O_2_, there was a sharp increase in mitophagy index and a decrease in the number of cells after 2 h (Fig. 7D, E) and at 4 h. In LAAO^WT^-treated samples, there was a 50% reduction in cell numbers (Fig. 7E). Thus, when treated with wild-type LAAO, a smaller number of cells have a very high mitophagy index. We surmise that mitophagy is a cellular response to counteract the impairment of mitochondria function by LAAO and thus elicit elimination via organelle quality control.

**Fig. 8:**
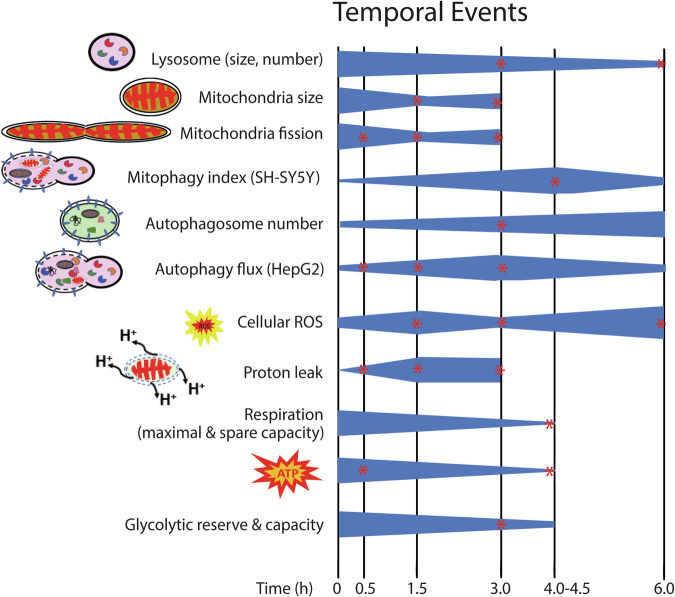
Summary of temporal events triggered by treatment with *B. atrox* LAAO^WT^. Mapping the changes of various cellular processes during LAAO envenomation. Quantitative morphometry was used to assess: (i) size and numbers of mitochondria and lysosomes (Figs. 3–5) and (ii) autophagosome numbers (Fig. 2). Functional assays evaluated autophagy flux index (Fig. 2), mitophagy index (Fig. 7), oxidative stress species (cellular ROS (Fig. 1)). Mitochondrial functions and energy generation (Fig. 6) are shown as proton leak, mitochondria respiration (maximal and spare capacity), ATP production, and glycolytic capacity and reserve. Normal keratinocytes were used in most assays. HepG2 cells expressing tandem mCherry-EGFP-LC3 and SH-SY5Y cells expressing mt-Keima were used as well-established models for autophagy flux index and mitophagy index, respectively. The symbol * denotes statistical significance, and *P*-values can be found in the respective figures.

All results put together (Fig. 8) showed a temporal segregation of the various cellular events. Despite normalization of LAAO EC_50_ for each cell type and condition when performing the experiments, distinct assay sensitivities and cell type-specific properties may affect the timing when a change in cellular events is detected. Nevertheless, a comparative analysis of phenotypes in a single cell type is feasible. Furthermore, while the timeframe might shift, key results confirmed among cell lines indicate the phenotype reproducibility across different cell types.

## Discussion

Here, we contribute to the conceptual understanding of the cellular events leading to tissue dysfunction triggered by a snake venom toxin, LAAO. While much is known about LAAO biochemistry and its ability to kill cells, the precise cellular mechanisms underpinning LAAO cytotoxicity are unclear. We identify unappreciated cellular processes disrupted by LAAO prior to induction of cell death and necrosis [18]. The reduced cell fitness induced by LAAO is catalysis-dependent and results from coordinated damage in mitochondria and lysosomes (perturbed organelle morphology, function, and clearance). Our novel finding in multi-organelle damage results in the shutdown of cellular metabolism and energy production prior to cell death.

The use of recombinant LAAO (glycosylated, soluble, and active) demonstrates unequivocally the relative contribution of essential residues for its enzymatic catalysis, which has been controversial in the past [39, 41, 47, 48]. Mutation R90A or Y372A strongly impairs LAAO^WT^ activity in vitro, consistent with the prediction of R90 as a cofactor (FAD) binding and cooperation with Y372 in substrate interaction. Glycosylation at residue N172 has been indirectly shown as relevant for catalysis [30]. LAAO^N172A^ mutant is indeed inactive, which may result from N-linked glycosylation contribution to maintain protein stability [49] or to regulate the exit of H_2_O_2_ from the catalytic pocket [39]. Thus, LAAO residues R90, N172 and Y372 have key catalytic functions, whereas other proposed amino acids are not essential (H223) or reduce (R322) activity. The latter two residues do not have a well-established role [39, 41].

Recombinant LAAO^WT^ cellular phenotypes reproduce those observed with native LAAO treatment [18]: higher levels of reactive oxygen species (ROS) and number of autophagosomes. We think it is unlikely that the higher autophagosome levels result from oxidative stress or amino acid modification or catabolism [27], as both wild-type and catalytic dead LAAO increase autophagosome numbers. Instead, we demonstrate that LAAO^WT^ attenuates autophagy flux index (i.e., autophagosome clearance) in a catalysis-dependent manner. An adaptive cellular response to oxidative stress generated by LAAO may promote autophagy flux via (i) altered catabolism of amino acids necessary to produce antioxidants (e.g., glutamine) or (ii) unbalancing the homeostasis of LAAO amino acid substrates, some of which (i.e., leucine) are key regulators of the amino acid-sensing machinery [27]. Furthermore, the byproducts of LAAO-dependent oxidation (H_2_O_2_ and ammonia) can themselves contribute to lysosomal defects [26, 50] and potentially impair cargo degradation, lysosome acidification and/or fusion with autophagosomes.

First, LAAO^WT^ treatment strongly decreases lysosome size and number, which limits the availability of degradative enzymes and reduces membrane area for the appropriate regulation of amino acid sensing by mTORC1 complexes [51]. Second, internalized LAAO^WT^ partially co-localizes with lysosomes, indicating that LAAO is at the right place to participate in lysosomal dysfunction. Such co-localization is novel for a snake toxin, and consistent with the preferential localization of its homolog IL4I1, a mouse B lymphocyte LAAO [52]. At the lysosomal acidic compartment, both enzymes have the optimal pH for their activity [52]. As lysosomes also participate in signaling, exocytosis, and nutrient sensing [53, 54], extensive lysosomal impairment by LAAO may also substantially damage cell homeostasis.

In addition to degrative processes, we find that LAAO^WT^ cytotoxicity (i) impairs mitochondria dynamics, the coordinated fission and fusion events [55, 56], and (ii) alters mitochondrial shape, size, and distribution in keratinocytes. Interference with mitochondrial function appears to be a main feature of native LAAOs from different snake venoms (altered mitochondrial membrane potential and Cytochrome C release) [17, 33, 57]. Furthermore, modulation of mitochondrial dynamics by oxidative stress and amino acid depletion is observed in other model systems [58] and is likely to contribute to LAAO-induced mitochondrial defects. Thus, LAAO interferes with fundamental cellular processes that have a domino effect in shutting down cell viability.

We predict that the impairment of mitochondrial dynamics by LAAO may also damage energy generation and consumption [59]. The mitochondrial network is highly dynamic and intimately associated with optimal bioenergetic capacity and the regulation of cellular signaling and metabolism [23]. The strong perturbation of mitochondria dynamics by LAAO^WT^ is accompanied by a shutdown of cell respiration and energy production, which severely restricts cell recovery. The rate of glycolysis and non-mitochondrial respiration over time is similar in controls and LAAO^R90A^. In contrast, LAAO^WT^ strongly reduces basal respiration and maximal mitochondrial capacity, thereby limiting ATP production. Under these conditions, cells reduce their ability to respond to energetic demand (spare capacity depletion) and adapt metabolically by using their glycolytic reserve to maintain glycolysis rate unchanged. In addition, LAAO^WT^ treatment prevents switching energy sources (glycolysis to oxidative phosphorylation) and reduces non-glycolytic acidification. Therefore, LAAO^WT^ impairs an adaptive energetic response that would facilitate cell recovery from mitochondria dysfunction.

Oxidative stress may trigger mitochondrial fission and mitophagy [23, 60], and indeed, mitophagy is stimulated by LAAO^WT^ exposure to remove damaged and smaller-sized mitochondria. In mt-Keima-expressing SH-SY5Y cells, fusion of mitochondria-containing autophagosomes with lysosomes occurs, but not their degradation. The attenuated clearance of mitochondria in SH-SY5Y cells in the presence of LAAO^WT^ is consistent with the inhibition of autophagy flux shown in HepG2 cells. The reproducibility of the lysosomal and mitochondrial defects in different cell types is significant for our understanding of the envenomation process.

In summary, our work builds on current knowledge of LAAO biochemistry and reveals unprecedented molecular and cellular mechanisms triggered by snake venom LAAO (Fig. 8). The mapped cellular phenotypes are temporally distinct, cooperatively reduce cell survival, and are likely to be interdependent. In the keratinocyte model, higher levels of proton leakage and increased mitochondria fission are the first detected events after LAAO treatment in keratinocytes, followed by increased oxidative stress. Later, an increase in the number of autophagosomes coincides with the reduced lysosome size and numbers. Such lysosomal defects contribute to impairment of organelle quality control and clearance, via inhibition of autophagy flux and mitophagy in different cell models (HepG2 and SH-SY5Y models, respectively). Alongside these phenotypes, there are profound changes in metabolic rates, energy production and consumption, which further compromise cell fitness. Thus, the inability to degrade and recycle damaged organelles in the presence of LAAO^WT^ is highly likely to synergize with oxidative stress and energetic impairment during cytotoxicity. In future studies, our in-depth data obtained in primary cells should be validated in animal models to move toward identifying therapeutic approaches.

Overall, our study provides a framework that is instrumental to enhance the mechanistic understanding of toxin action on its own right and thus, a step change in our knowledge of the envenomation process. The early, multiple organelle impairment caused by LAAO exposure highlights points of future interventions in in vivo models to restore tissue homeostasis and renewal in an envenomation context.

## Supplementary information

Supplementary figures and legends

TableS1

Supplementary File S1

## Data Availability

All data supporting this study are presented in this published article and in its Supplementary information files.
