# Supplementary material for: Functional analyses and integrated mechanisms of cellular destruction by L-amino acid oxidase
Source: Cell Death Dis. 2025 Nov 20;17(1):48. doi: 10.1038/s41419-025-08187-7 (PMC12811378; doi:10.1038/s41419-025-08187-7)
Supplement: Supplementary file 1 — Supplementary figures and legends [file 41419_2025_8187_MOESM1_ESM.docx]

SUPPLEMENTAL INFORMATION

for

**Functional analyses and integrated mechanisms of cellular destruction by L-amino acid oxidase**

Krisna Prak, Christin Luft, Eliona Tsefou, Carlos Chávez-Olórtegui, Janos Kriston-Vizi, Robin Kettler, Vania M. M. Braga

**This file includes:**

Supplemental Figures S1 to S5 and legends

**Other Supplemental information for this manuscript include the following:**

Table S1 lists primer sequences used for cloning

File S1 reports Sample Size and Replicates, Detail on Data Distribution, Effect Size Reporting, and Handling of Time-Dependent Data.


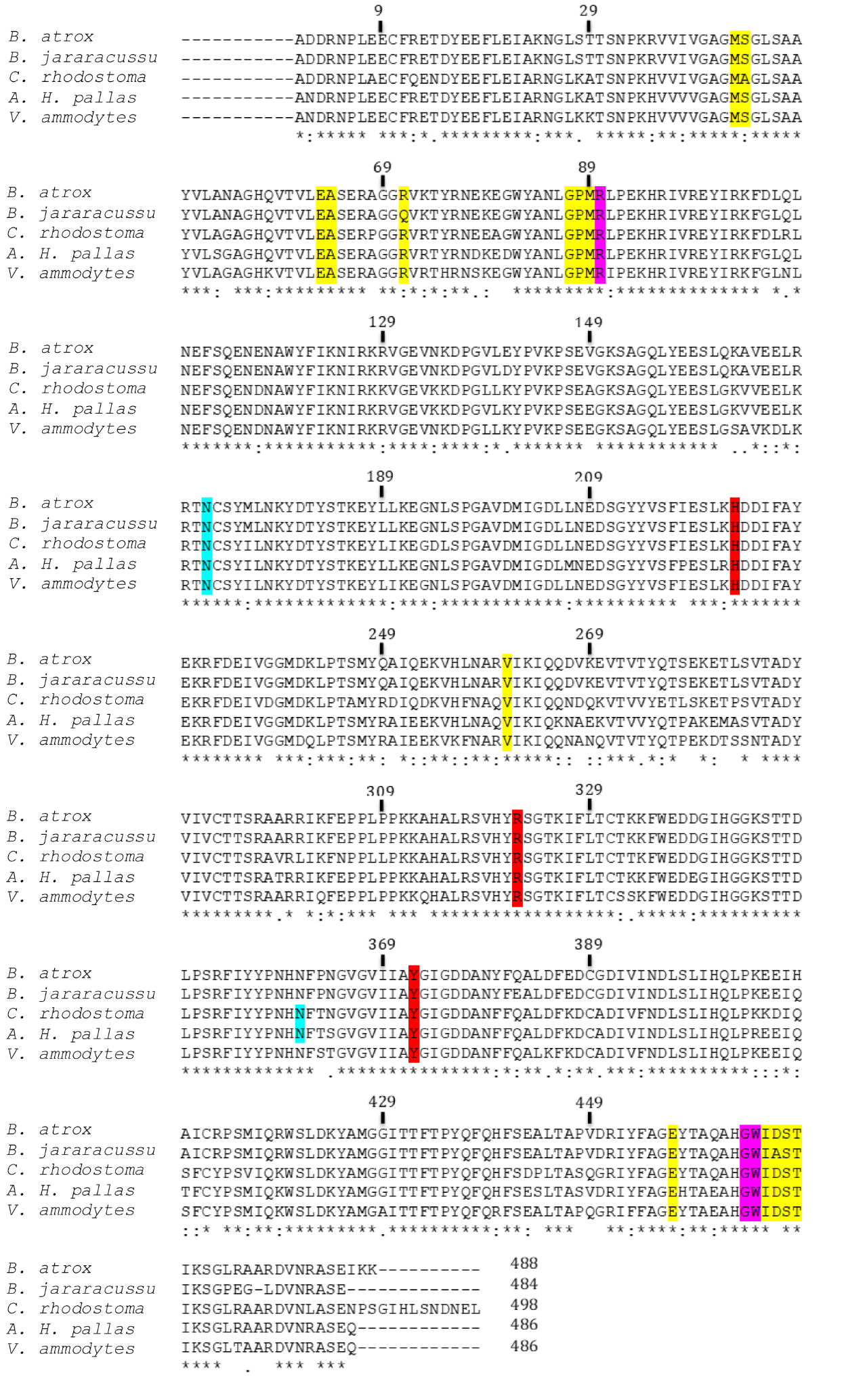


**Fig. S1**. **Alignment of the amino acid sequences of known snake venom LAAO structures.**

LAAO sequences from the species *B. atrox* (5TS5)*, B. jararacussu* (PDB 4E0V), *C. rhodostoma* (PDB 2IID)*, A. H. pallas* (PDB 1REO)*, and V. ammodytes* (PDB 3KVE) were aligned using Clustal Omega (1.2.1) multiple sequence alignment. Predicted cofactor FAD binding residues are shaded in yellow; N-linked glycosylation residues are shaded in cyan, substrate binding residues are shaded in red, FAD and substrate binding residues are shaded in pink. Amino acid substitutions are denoted as: * (identical residues); : (conserved substitutions); . (semi-conserved substitutions).


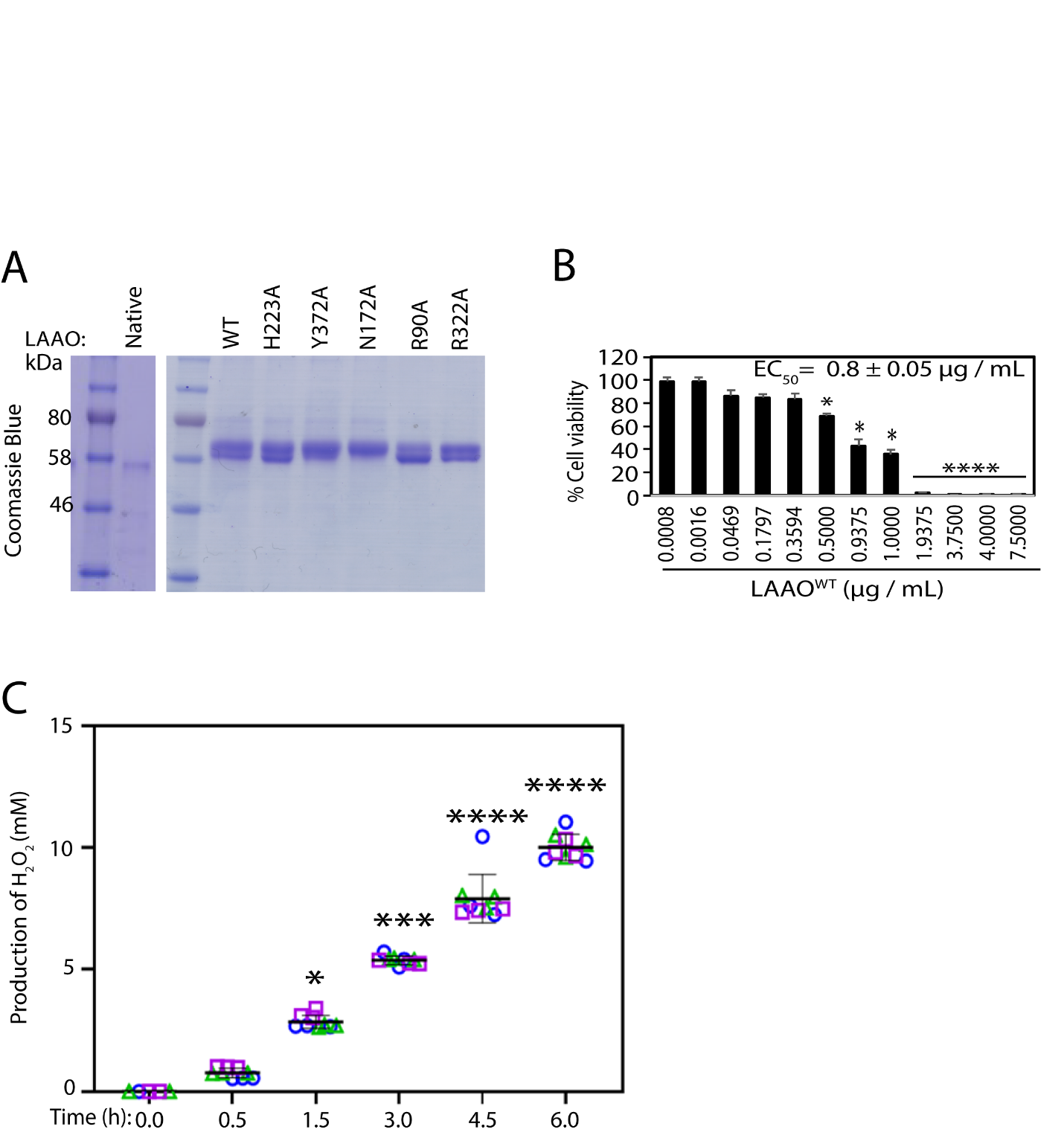


**Fig. S2**. ***B. atrox* LAAO^WT^ induces cell cytotoxicity**.

**A**, **Purification of recombinant LAAO-6xHis wild-type (WT) and mutants.** Recombinant proteins were separated in SDS−PAGE and stained with Coomassie brilliant blue. Native LAAO purified from *B. atrox* venom was used as control. Purified recombinant LAAOs has a lower mobility than native LAAO (UniProtKB: P0CC17; 488 aa, approximately 55.5 kDa), due to additional amino acid residues for targeting for secretion and His-tag sequence. Glycosylation differences between mammalian (HEK293 cells) and reptilian cells may also contribute to its distinct electrophoretic mobility. **B**, **Calculation of Effective concentration of LAAO^WT^ (EC_50_) in keratinocytes**. Cells were incubated with different concentrations of LAAO for 24 h and the metabolic cell fitness was analysed using Alamar Blue. **C, Determining the amount of H_2_O_2_ produced by LAAO^WT^** when added to the medium in the absence of cells. LAAO^WT^ (28 nM) was added to DMEM phenol-free supplemented with 1% FCS and Glutamax and incubated at 37 °C, 5% CO_2_ for a period from zero to 6 h. The amount of H_2_O_2_ content in the samples were calculated using a H_2_O_2_ standard curve. Results are displayed as mean ± standard deviation. N=3.


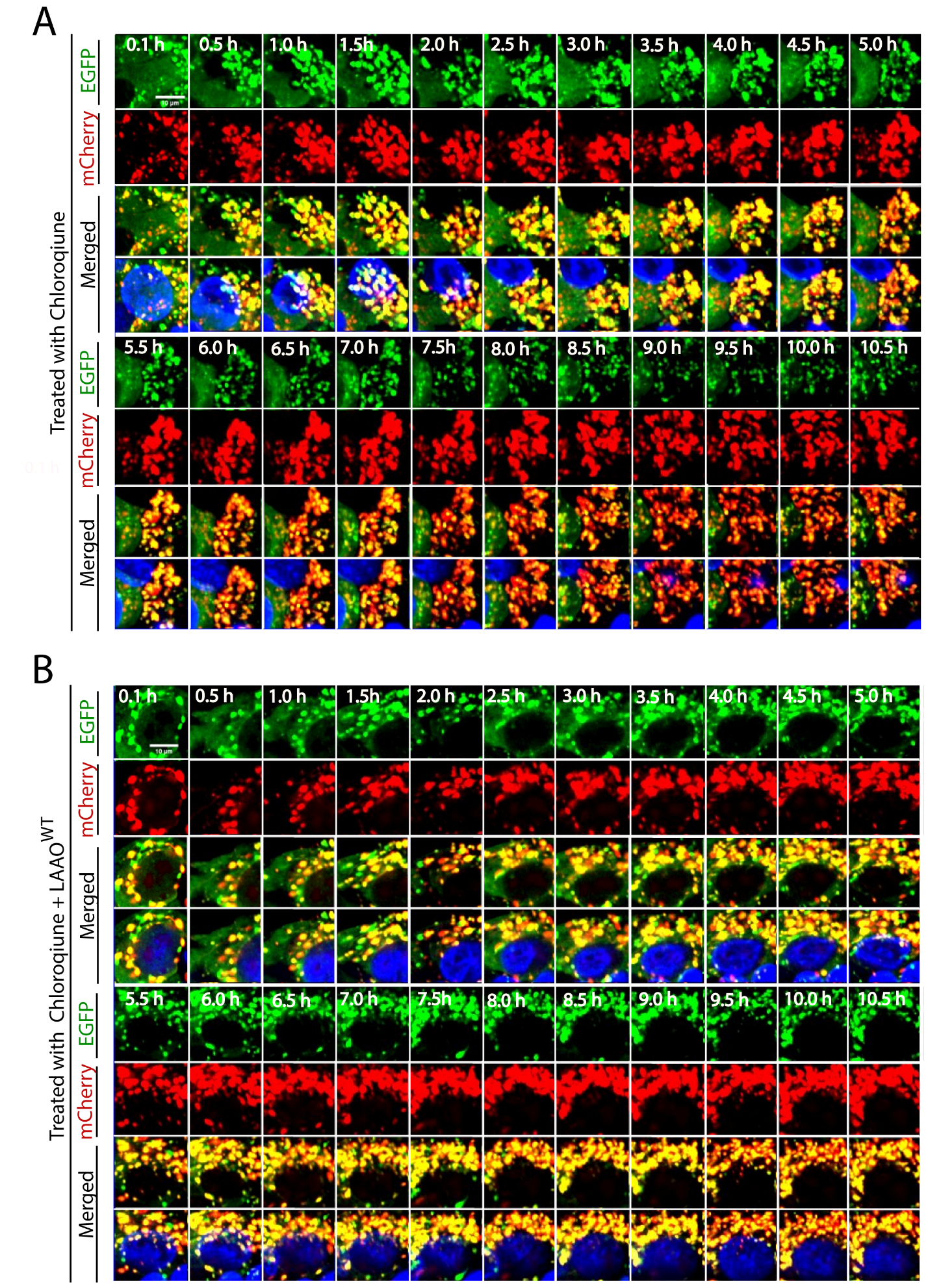


**Fig. S3**. **HepG2 cells stable expressing tandem mCherry-EGFP-LC3 and treated with chloroquine show inhibition of autophagy flux and this effect is sustained by treatment with** **LAAO^WT^.**

HepG2 cells stable expressing tandem mCherry-EGFP-LC3 were treated with 10 µM of chloroquine or 10 µM of chloroquine and 1X EC_50_ (42 nM for HeG2 cells) of LAAO^WT^ during a time course. Confocal live images show for each sample: EGFP-LC3 fluorescence (top rows), mCherry-LC3 fluorescence (middle rows) and the merged image (bottom rows). Nuclei were stained with low concentration of Hoechst 33342 (blue), and images captured every 30 min. The images complement the data shown in Figure 4A-C and were collected in parallel with the same settings. Scale bar: 10 µm. N=3.


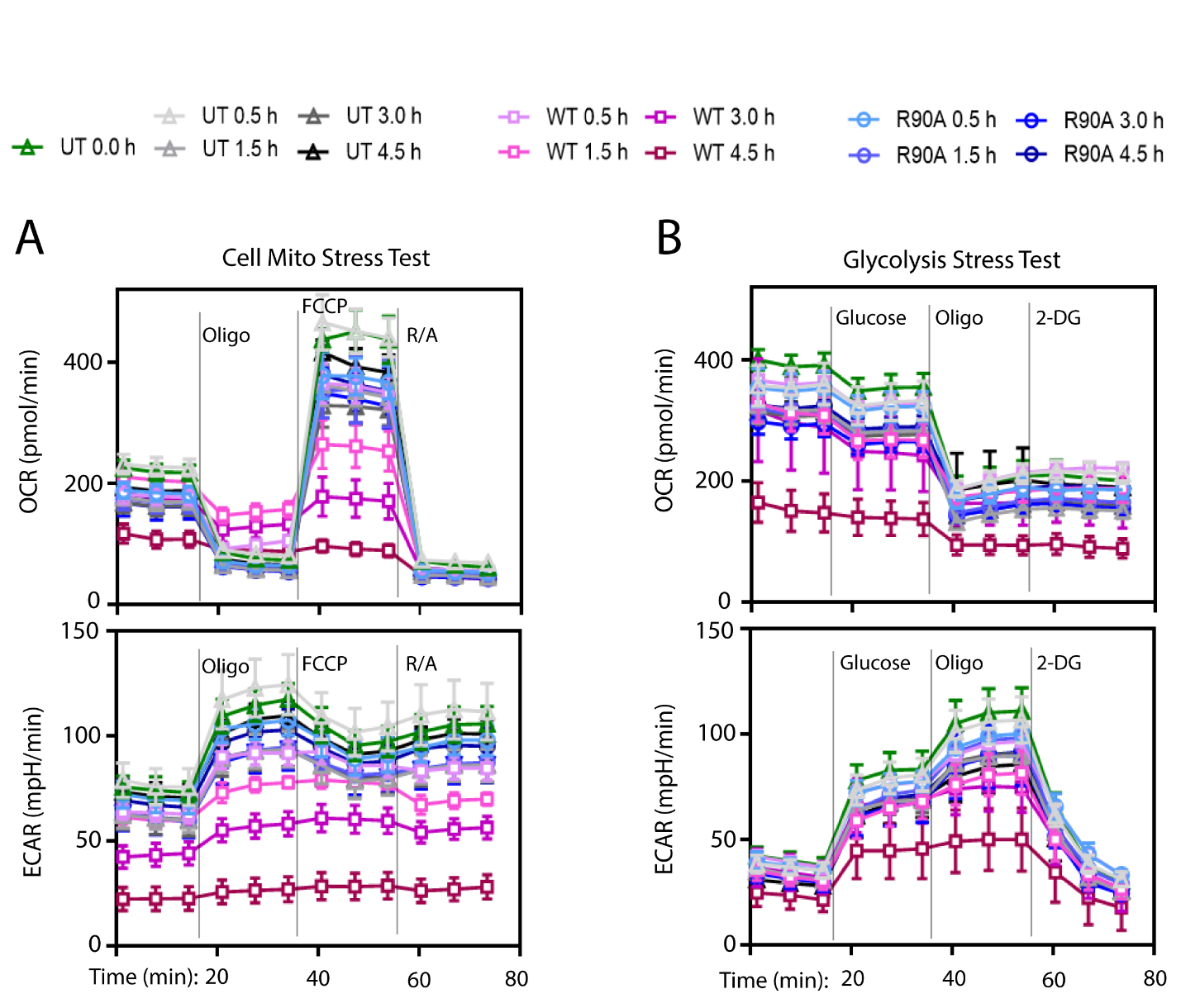


**Fig. S4.** **Profiles of oxygen consumption rate (OCR) and extracellular acidification rate (ECAR) during mitochondria or glycolysis stress test following incubation with *B. atrox* recombinant LAAO^WT^ or LAAO^R90A^.**

Keratinocyte were left untreated (UT) or incubated with 2 X EC_50_ (73 nM for SeaHorse experiments) LAAO^WT^ or LAAO^R90A^ for 0.5, 1.5, 3.0, and 4.5 h. Cells were then subjected to mitochondrial stress test (A) or Glycolysis stress test (B)**.** The profiles shown complement data shown in Figure 6. **A**, Mitochondrial stress test when challenged by oligomycin (oligo, an ATP synthase inhibitor), carbonylcyanide-p-trifluoromethoxyphenyl hydrazine (FCCP, an electron transport chain uncoupler) or rotenone/antimycin A (R/A, inhibitors of electron transport chain complex I and III). The addition of each chemical at a particular time is indicated as vertical grey lines. **B**, Glycolysis stress test was performed in the presence of glucose (fuel for glycolysis), oligomycin or 2-deoxyglucose (2DG, an inhibitor of glycolysis). N=3.


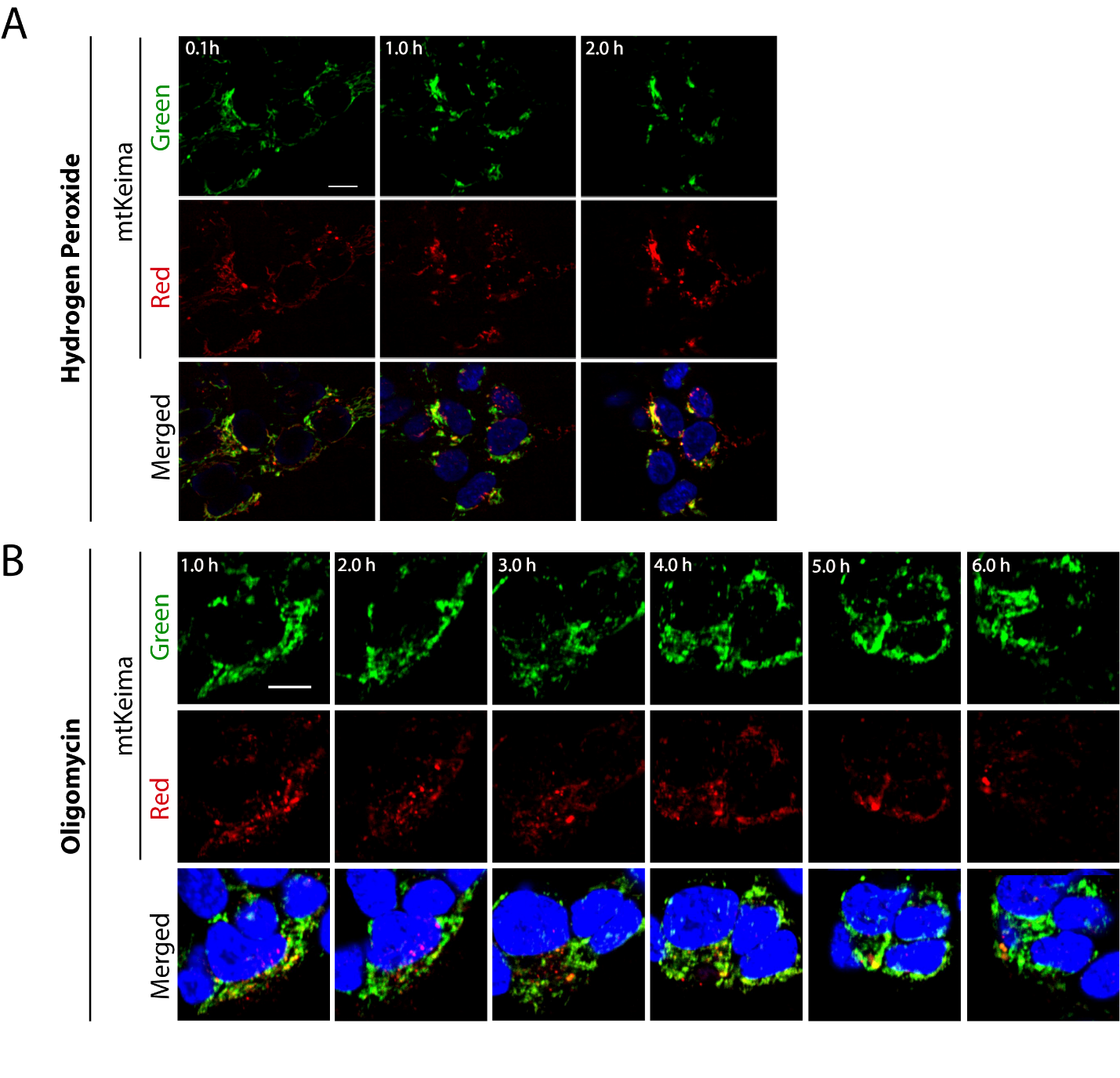


**Fig. S5**. **Mitophagy responses of SH-SY5Y cells stably expressing mtKeima to hydrogen peroxide (A) or oligomycin (B).**

Confocal live imaging of cells shows for each treatment: cytoplasmic mtKeima (green, top row), lysosomal mtKeima (red, middle row) and nucleus (blue, bottom row). The images complement the data shown in Figure 7C and were collected in parallel with the same settings. Scale bar: 10 µm. N=3.
